# Characterization of cassava ORANGE proteins and their capability to increase provitamin A carotenoids accumulation

**DOI:** 10.1371/journal.pone.0262412

**Published:** 2022-01-07

**Authors:** Angélica M. Jaramillo, Santiago Sierra, Paul Chavarriaga-Aguirre, Diana Katherine Castillo, Anestis Gkanogiannis, Luis Augusto Becerra López-Lavalle, Juan Pablo Arciniegas, Tianhu Sun, Li Li, Ralf Welsch, Erick Boy, Daniel Álvarez

**Affiliations:** 1 HarvestPlus, c/o The Alliance of Bioversity International and the International Center for Tropical Agriculture (CIAT), Cali, Colombia; 2 The Alliance of Bioversity International and the International Center for Tropical Agriculture (CIAT), Cali, Colombia; 3 Robert W. Holley Center for Agriculture and Health, USDA-ARS, Cornell University, Ithaca, New York, United States of America; 4 Faculty of Biology II, University of Freiburg, Freiburg, Germany; 5 HarvestPlus, International Food Policy Research Institute, Washington, DC, United States of America; University of Tsukuba, JAPAN

## Abstract

Cassava (*Manihot esculenta* Crantz) biofortification with provitamin A carotenoids is an ongoing process that aims to alleviate vitamin A deficiency. The moderate content of provitamin A carotenoids achieved so far limits the contribution to providing adequate dietary vitamin A levels. Strategies to increase carotenoid content focused on genes from the carotenoids biosynthesis pathway. In recent years, special emphasis was given to ORANGE protein (OR), which promotes the accumulation of carotenoids and their stability in several plants. The aim of this work was to identify, characterize and investigate the role of OR in the biosynthesis and stabilization of carotenoids in cassava and its relationship with phytoene synthase (PSY), the rate-limiting enzyme of the carotenoids biosynthesis pathway. Gene and protein characterization of OR, expression levels, protein amounts and carotenoids levels were evaluated in roots of one white (60444) and two yellow cassava cultivars (GM5309-57 and GM3736-37). Four OR variants were found in yellow cassava roots. Although comparable expression was found for three variants, significantly higher OR protein amounts were observed in the yellow varieties. In contrast, cassava *PSY1* expression was significantly higher in the yellow cultivars, but PSY protein amount did not vary. Furthermore, we evaluated whether expression of one of the variants, *MeOR_X1*, affected carotenoid accumulation in cassava Friable Embryogenic Callus (FEC). Overexpression of maize *PSY1* alone resulted in carotenoids accumulation and induced crystal formation. Co-expression with *MeOR_X1* led to greatly increase of carotenoids although *PSY1* expression was high in the co-expressed FEC. Our data suggest that posttranslational mechanisms controlling OR and PSY protein stability contribute to higher carotenoid levels in yellow cassava. Moreover, we showed that cassava FEC can be used to study the efficiency of single and combinatorial gene expression in increasing the carotenoid content prior to its application for the generation of biofortified cassava with enhanced carotenoids levels.

## Introduction

Vitamin A is essential for vision and cell differentiation and its deficiency is the main cause of preventable blindness, cause development disorders, and impairs the immune system [1, 2]. Vitamin A deficiency persists as a major public health problem globally, and disproportionately affects preschool children and pregnant women in low- and middle-income countries [3–5]. Thus, the first and most important step to prevent primary vitamin A deficiency is the regular consumption of vitamin A-rich foods [6]. Plant foods provide vitamin A precursors, also known as provitamin A (pVA) carotenoids. Carotenoids are natural lipophilic isoprenoids that are involved in photosynthesis, provide pigmentation to a wide range of plant tissues, and serve as precursors for the formation of phytohormones and signaling precursors essential for plant homeostasis [7, 8]. β-Carotene is one of the most abundant carotenoids in nature and along with α-carotene and β-cryptoxanthin are the main carotenoids in plants with pVA function [9, 10].

Cassava (*Manihot esculenta* Crantz) is a native crop to Central and South America [11]. It is highly appreciated for its ease of agronomic handling, high productivity, and tolerance to poor soils and drought [12], thus it is a major crop in low- and middle-income countries in the tropics [13, 14]. The leaves and roots are used for human consumption, livestock feed, and starch production [15–17]. In Africa, cassava is the second most important staple food in terms of calories supply [18]. However, white cassava roots are a poor source of micronutrients such as iron, zinc, and pVA carotenoids, and only very few yellow-root cultivars synthesize and store carotenoids [19]. Thus, there is a need to increase the content of pVA carotenoids for enhancing the nutritional quality of agronomically preferred cassava varieties that can be incorporated into diets and contribute to alleviating vitamin A deficiency.

Biofortification is the process of increasing minerals and vitamins in food crops through conventional breeding, genetic engineering, and agronomic practices [20]. It is a complementary strategy to other interventions such as the promotion of a diverse diet, food fortification, or supplementation. Biofortification seeks to mitigate nutritional deficiencies through the consumption of staple food crops [21]. It is considered economically cost-effective and sustainable, in addition to having the capacity to reach people with limited access to other nutritional interventions [22, 23].

To increase the content of pVA carotenoids in cassava, conventional breeding has focused on QTLs related to the carotenoids biosynthesis pathway [24]. Phytoene synthase (PSY) is the first specific enzyme in the carotenoid biosynthesis pathway and is considered as rate-limiting for carotenoid biosynthesis. Cassava has three *PSY* genes, of which *PSY1* and *PSY2* are highly abundant in leaves while *PSY2* transcripts dominate in roots [25]. *PSY3* expression is absent in all tissues analyzed so far but suggested to be involved in carotenoid biosynthesis for apocarotenoid signal compounds as shown for the tomato homologue [26]. *PSY1* is more responsive to abiotic stress than *PSY2* while *PSY2* shows higher abundance in petals and roots, suggesting some specificity for carotenoid biosynthesis in non-green tissues. However, there is no association between the expression levels of any cassava *PSY* paralog and carotenoid accumulation in cassava varieties [27] while a SNP in *PSY2* explained most of the carotenoid variations. In fact, it was shown that this polymorphism increased the enzymatic activity of recombinant PSY enzymes by 3-fold and suggested that PSY activity determined the total carotenoid content in cassava roots. Accordingly, white cassava genotypes transformed with the bacterial *PSY* gene *CRTB* produced increases of up to 22 μg/g (DW) of total carotenoids and 7 μg/g (DW) of β-carotene, the most abundant carotenoid in cassava roots [27]. However, the fact that mainly carotene intermediates like phytoene accumulated, unraveled carotene desaturation as a subsequent rate-limitation for β-carotene formation. When transformed with *CRTB* gene alone or in combination with the bacterial *phytoene desaturase* gene (*CRTI*) or with the upstream gene *1-deoxyxylulose 5-phosphate synthase* (*DXS*), the highest levels achieved were 60 μg/g (DW) of total carotenoids in cassava roots [28, 29].

Despite these high levels using transgenic approaches, those obtained by means of conventional breeding methods were not surpassed (70 μg/g DW) [30]. However, these varieties did not meet characteristics such as optimal size, cooking time, safety, and texture properties among others to make them available to the general population for their adoption [31] Hitherto, the highest levels of total pVA carotenoids reached by biofortified varieties with favorable adoption characteristics were up to 50 μg/g (DW) [32].

Carotenoids are subjected to enzymatic and more importantly to oxidative non-enzymatic degradation [33–35]. In cassava, postharvest processes such as storage conditions can produce significant losses of carotenoids of 20–50% [36, 37], while 5–95% may be lost upon cooking with different methods [19, 38–40]. Considering the only moderate carotenoid content achieved so far in edible cassava genotypes, and the losses caused by postharvest processes, the retention of pVA carotenoids can be low. The Estimated Average Requirement (EAR) of vitamin A for children and women of reproductive age is 275 μg and 500 μg according to the Institute Of Medicine [41]. Thus, even though considering a high conversion rate of pVA carotenoids into vitamin A of 5:1 in cassava, the final contribution of biofortified cassava varieties to the EAR could be modest at best [36, 40].

The lack of efficient molecular tools for carotenoid accumulation in cassava has hindered the development of cassava varieties with superior content of pVA carotenoids, as in carrot [42, 43], maize [44], or sweet potato [45, 46]. Thus, the identification of novel genes in cassava that can boost carotenoid accumulation and increase protection against degradation is critical to assuring a sufficient pVA supply by cassava to the diet.

ORANGE (OR) protein has gained increasing interest in recent years as it has several functions related to carotenoid accumulation and stabilization [47, 48]. The *OR* gene is not part of the carotenoid biosynthetic pathway and it was found originally from an orange-curd cauliflower (*Brassica oleracea*) mutant with a high content of carotenoids [49]. An increase in carotenoid content was observed alongside a higher storage capacity, but with no significant change in the expression of *OR* or *PSY* genes [50, 51]. In the cauliflower OR mutant, a mutation in the *OR* gene caused by a retrotransposon produces three in-frame splicing variants with partially altered molecular properties [51, 52]. In orange-fleshed melon, however, a SNP in the *OR* is responsible for large amounts of carotenoids accumulation [53], similarly in carrot [54].

OR is a holdase chaperone highly conserved among plants and can be localized in both the chloroplasts and the nucleus [55–58]. It is considered to induce chromoplast differentiation and carotenoid crystals formation, thus generating a sink for carotenoid storage in potato, Arabidopsis and melon [59–61]. It can also attenuate β-carotene metabolism, although it is yet unknown whether the expression of specific genes such as *β-carotene hydroxylase* (*BCH*) are altered to lead to ∝-carotene accumulation [44, 62–64]. Likewise, OR shown to physically interact with PSY in Arabidopsis, sweet potato, and cauliflower, leading to the post-translational regulation of this protein as well as mutual co-regulation with a consequent increase in the production of carotenoids [52, 56, 65]. The *OR* gene can also improve abiotic stress tolerance in potato and sweet potato possibly altering the abscisic acid (ABA) signaling pathway [65–67]. Additionally, OR in Arabidopsis can interact in the nucleus with transcription factors involved in chloroplast biogenesis and interacts with plastid division factor to interfere with chromoplast division [57, 68]. Some plants such as Arabidopsis and melon harbor OR-like proteins, which are homologous OR proteins with unique but partially redundant functions [56, 69].

The relevance of OR in the accumulation of carotenoids has been reported extensively upon the overexpression of the wild type or mutagenized *OR* genes from Arabidopsis, cauliflower, sweet potato, or melon in potato [60, 66], Arabidopsis [70], rice [71], maize [44], tomato [72], sweet potato [65, 67], and cauliflower [52], among other plants [73, 74]. However, sparse information is available on the effect of the *OR* on the accumulation of carotenoids in yellow cassava [29]. The aim of this study was to identify *OR* genes in cassava, investigate their correlation with carotenoids accumulation, and explore their potential to increase and stabilize carotenoid levels in cassava, using cassava *in vitro* tissues as a model system.

## Materials and methods

### Sequence analyses

The Phytozome database (https://phytozome.jgi.doe.gov/) was used to identify *OR* family members in the cassava genome using the coding sequence (CDS) of *OR* homologs from melon (*Cucumis melo*, accession no. A0A0D3MU50.1 and *Cucumis melo CmOR*-like, accession no. MELO3C024554), cauliflower (*Brassica oleracea*, accession no. A2T1U1.1), Arabidopsis (*Arabidopsis thaliana*, accession no. AT5G61670 and *Arabidopsis thaliana AtOR-like*, accession no. AT5G06130), and sweet potato (*Ipomoea batatas*, accession no. APG21184.1). The identity of the cassava amino acid sequences to homologous OR sequences in melon, cauliflower, Arabidopsis, and sweet potato was studied using BLAST (https://blast.ncbi.nlm.nih.gov/Blast.cgi).

The phylogenetic tree and corresponding alignment were performed with MEGA X [75]. A neighbor-joining method was selected with a bootstrap analysis using 1000 replicates. DNA and deduced amino acids of OR homologous sequence alignments as well as cDNA comparisons for SNP detection were carried out with Clustal Omega [76].

The Splign tool (https://www.ncbi.nlm.nih.gov/sutils/splign/splign.cgi) was used for computing the alignments of the cDNA sequences and determine size and relative position of exons [77].

Chloroplast transit peptides (cTP) of MeOR variants were predicted using the tool ChloroP [78].

Prediction analysis to identify transmembrane domains was carried out using Phobious (phobius.sbc.su.se) [79].

### Plant material

Two cassava (*Manihot esculenta* Crantz) genotypes with yellow root flesh (GM5309-57, “Y1,” and GM3736-37, “Y2”) and one genotype with white root flesh (60444, “White”) were grown at the International Center for Tropical Agriculture (CIAT, Palmira, Colombia). For each genotype, one storage root in three different plants was harvested at 11 months. Immediately afterwards, the roots were peeled, washed with 18 MΩ water, and scrubbed with RNase decontamination wipes free of RNases, DNases, and pyrogens (RNaseZapTM, Invitrogen). Once cleaned, the roots were wrapped in aluminum foil and placed in liquid nitrogen. The samples were shredded with a metallic food grater and ground to a fine powder using a mortar and pestle. The benchtop and materials were cleaned with RNAseZap wipes and abundant liquid nitrogen was used during the grinding process to prevent RNA degradation. The samples were stored at ‒80°C until use.

### RNA extraction and real time qRT-PCR analysis

Total RNA was extracted according to Behnam et al. [80] and integrity was determined by an Agilent 2100 Bioanalyzer. For cDNA synthesis, 2 μg of total RNA was treated with amplification grade DNase I (Invitrogen) to remove DNA contamination. The samples were reverse-transcribed with SuperScript^™^ III Reverse Transcriptase Kit, Random Hexamers, and RNaseOUT^™^ (Invitrogen) according to the manufacturer’s’ instructions. Real-time qRT-PCRs were performed using a PowerUp^™^ SYBR^™^ Green Master Mix (Applied Biosystems), along with gene-specific primers (S1 Table) in a QuantStudio 5 real-time PCR system (Applied Biosystems). Data were analyzed with the QuantStudio^™^ design and analysis software v1.5 (Applied Biosystems) using relative quantification [81]. Histidine and ubiquitin genes were used as reference genes [82, 83] and white-fleshed cassava genotype 60444 was used as a calibrator. The efficiency of the reaction was calculated with the elaboration of standard curves by means of serial dilutions. Biological triplicates and technical duplicates were used.

### Sequencing

The cDNAs from roots of genotype Y1 were sequenced by Macrogen, Inc. (Seoul, South Korea) with gene-specific primers (S2 Table) to obtain the CDS of *MePSY1*, *MePSY2*, *MeOR_X1*, and *MeOR_X2*. Sequencing quality was checked with BioEdit 7.2 Sequence Alignment Editor [84].

### SNP identification and differential diversity analysis

SNPs in the sequences of Y1 were identified by means of a comparison of the cDNA sequences of the genes under study to the corresponding DNA sequences from the Reference Genome Sequence v6.1 of cassava [85]. The allelic frequencies of SNP loci were investigated in a population of 330 cassava varieties from CIAT’s GeneBank, with diverse root flesh colors and carotenoid contents. For each background sample, raw whole genome sequence reads (average 10X coverage, 2x150 bp length) were aligned with Burrows-Wheeler Aligner [86] to the Reference Genome Sequence v6.1 of cassava. Variations were called with GATK and filtered with vcftools [87].

### Protein extraction and western blot analysis

The extraction and quantification of total protein were carried out from 1 g of frozen cassava roots according to Maass et al. [88] with modifications of Cuellar et al. [89]. Proteins were resolved on a 12% SDS-polyacrylamide gel and blotted to 0.45 μM PVDF membranes (ThermoFischer). The blotting was conducted in a Mini Trans-Blot^®^ Cell (Bio-Rad) apparatus. Membranes were blocked with 5% skim milk powder in Tris-buffered saline, washed, and incubated in Tris-buffered saline plus 0.1% Tween 20 with either the polyclonal antibodies anti-PSY [56] or anti-OR [51]. After washing, the membranes were incubated for 1 h with a horseradish peroxidase- conjugated goat anti-Rabbit IgG antibody (Invitrogen, Cat.# G-21234) in a 1% skim milk solution in Tris-buffered saline plus 0.1% Tween 20. The membranes were washed and immunoblots were developed with Pierce^™^ ECL Western Blotting Substrate (ThermoFischer). After striping the membrane with peroxidase [90], an anti-actin antibody (Sigma-Aldrich, Cat.#A0480) and a horseradish peroxidase-conjugated goat anti-Mouse IgG antibody (Invitrogen, Cat.# G-21040) were used to reprobe the immunoblot. Protein signals were quantified with ImageJ [91].

### Carotenoids extraction and quantification

The extraction of carotenoids from cassava roots was carried out according to Jaramillo et al. [92] using 3 g of lyophilized roots. Carotenoids from Friable Embryogenic Callus (FEC) were extracted according to [93] using 10 mg of lyophilized FEC.

### Friable Embryogenic Callus (FEC) culture

*In vitro* cassava plants (variety 60444) were propagated by 2-cm cuttings on medium ME001 and were cultured under 16/8 photoperiod at 28°C in controlled conditions. After 35 days, the plantlets were defoliated and axillary buds from nodal explants were dissected and placed in liquid embryo induction medium (L-EIM) for 21 days at 28°C in the dark. Then, after the formation of primary embryos, the tissue was transferred to solid embryo induction medium (S-EIM) at 28°C under low light conditions to produce secondary embryos for 29 days [94]. Once the secondary embryos were formed, they were transferred to medium ME001 for maturation and cluster formation. These clusters were used to produce FEC according to Taylor et al. [95].

### Plasmid construction

Plasmid containing cDNA for *ZmPSY1* [96] was provided by Dr. Ralf Welsch (University of Freiburg, Germany). The backbone plasmids *pMDC123* [97] and *pCAMBIA 1305*.*2* [98] for cassava transformation were kindly provided by Dr. Paul Chavarriaga (CIAT’s Advanced Breeding Platform). The construction of the vector *pCAMBIA1305*.*2-MeOR_X1* was carried out by cloning the CDS of *MeOR_X1* previously obtained from cDNA of roots of genotype Y1 after PCR amplification and purification, into the plasmid *pCAMBIA1305*.*2*. To produce the vector *pMDC123-ZmPSY1*, the CDS of *ZmPSY1* was subcloned into the plasmid *pMDC123*. The construction of all vectors was carried out by the company LakePharma, Inc. (Irving, TX, USA).

### FEC transformation and propagation

*Agrobacterium tumefaciens* strain LBA4404 containing the plasmid *pMDC123-ZmPSY1* or *pCAMBIA1305*.*2-MeORX1* was used to transform FEC of cassava variety 60444 following the protocol of Bull et al. [99], with modifications by Brand et al. [94], to produce transformants containing either *ZmPSY1* or *MeOR_X1*, respectively. Additionally, FEC was also co-transformed with *ZmPSY1* and *pCAMBIA1305*.*2-MeORX1* to produce the transformant *MeOR_X1* + *ZmPSY1*. Transformed FEC was subcultured in Gresshoff and Doy (GD) containing 20 g/L sucrose, CuSO_4_ 200 μM, and 8 g/L Agar, supplemented with Picloram 12 mg/L and Cefotaxime 100 mg/L following the protocol of Taylor et al. [95], with modifications of Brand et al. [94]. After transformation, thirty to fifty individual cell lines were obtained for each construct, which were continuously grown under selective medium. The following selection scheme was applied: control: no selection; *ZmPSY1*: phosphinotricin 1 mg/L; *MeOR*: hygromycin 15 mg/L; *ZmPSY1 + MeOR*: phosphinotricin 1 mg/L and hygromycin 15 mg/L. Samples were grown for two months subjected to two cycles of selection under tender light at 28 ºC, followed by one month under dark conditions at the same temperature. Besides the ability of cell lines to grow on selective medium, the appearance of color, yellow to orange, was the second criteria for selection. Individual cell lines were pooled to form larger cell clusters that were later used for RNA extraction.

### Protoplast isolation and microscopic analyses

Proliferated FEC from mixed single- or two-plasmid transformation events was disaggregated in 2 mL of liquid culture media TM2G modified from Sofiari et al. [100] and 2 mL of enzymatic solution. The tissue was digested overnight in dark at 28°C and 30 rpm. The solution with digested FEC was filtered through three Myracloth layers, followed by two washes with WI solution and centrifugation at 1000 rpm for 5 minutes. The pellet was resuspended in 1 mL of TM2G for microscopy visualization. The sample was loaded in Neubauer chamber and protoplasts images were taken at 40X objective with microscope Leica DM500 equipped with Leica ICC50 HD and LAS EZ software.

### Data analysis

Data were analyzed using univariate analysis of variance and the results were expressed as the mean ± standard deviation. The comparison of means was performed based on paired *t*-tests with a significance level of *p*<0.05 in SAS (v9.3).

### Accession numbers

The *OR* sequence data used in this article can be found in the GenBank database with the following accession numbers: *OsOR*, XP_015622925.1; *ZmOR*, ACN31016; *SbOR*, XP_002452827; *CmOR*, A0A0D3MU50.1; *CmOR-like*, MELO3C024554; *AtOR*, AT5G61670; *AtOR-like*, AT5G06130; *BoOR*, A2T1U1.1; *SlOR*, NP_001315338.1; and *IbOR*, APG21184.1. The sequences of *MeOR_X1* and *MeOR_X2* were submitted to GenBank and the following accession numbers were assigned: MW246837 for *MeOR_X1* and MW246838 for *MeOR_X2*.

## Results

### Sequence analysis

Five putative *OR* genes were found in the Phytozome database, three on chromosome 14 (named *MeOR_X1*, *MeOR_X1*.*2*, and *MeOR_X3*), one on chromosome 6 (*MeOR_X2*), and one on chromosome 9 (*MeOR_X4*; Table 1). The corresponding molecular weight (MW) of the proteins was predicted and an alignment in BLAST was performed to determine their identity with the OR proteins from melon, cauliflower, Arabidopsis, and sweet potato. The predicted proteins of *MeOR_X1* and *MeOR_X2* presented a high identity with OR proteins from other plants. Conversely, the predicted *MeOR_X4* showed a high identity with OR-like proteins.

**Table 1:** Identification of cassava OR variants, deduced protein MW, and identity with OR proteins from various plant species.

Transcript name	Variant name	Chromosome	Predicted protein (kDa)	Identity with CmOR (%)	Identity with CmOR-like (%)	Identity with BoOR (%)	Identity with IbOR (%)	Identity with AtOR (%)	Identity with AtOR-like (%)
Manes.14G040800.1	MeOR_X1	14	34.4	84.21	67.15	75.16	78.27	76.27	63.27
Manes.14G040800.2	MeOR_X1.2	14	27.4	72.48	66.67	73.47	74.07	73.98	65.37
Manes.06G130100.1	MeOR_X2	6	41.2	77.37	69.77	72.90	79.68	75.95	67.46
Manes.14G040300.1	MeOR_X3	14	29.8	64.94	59.34	64.97	67.41	65.82	56.41
Manes.09G053200.1	MeOR_X4	9	34.7	66.55	82.48	65.33	69.03	68.25	79.46

Cm, *Cucumis melo*; Bo, *Brassica oleracea*; At, *Arabidopsis thaliana*; Ib, *Ipomoea batatas*.

**Fig 1:**
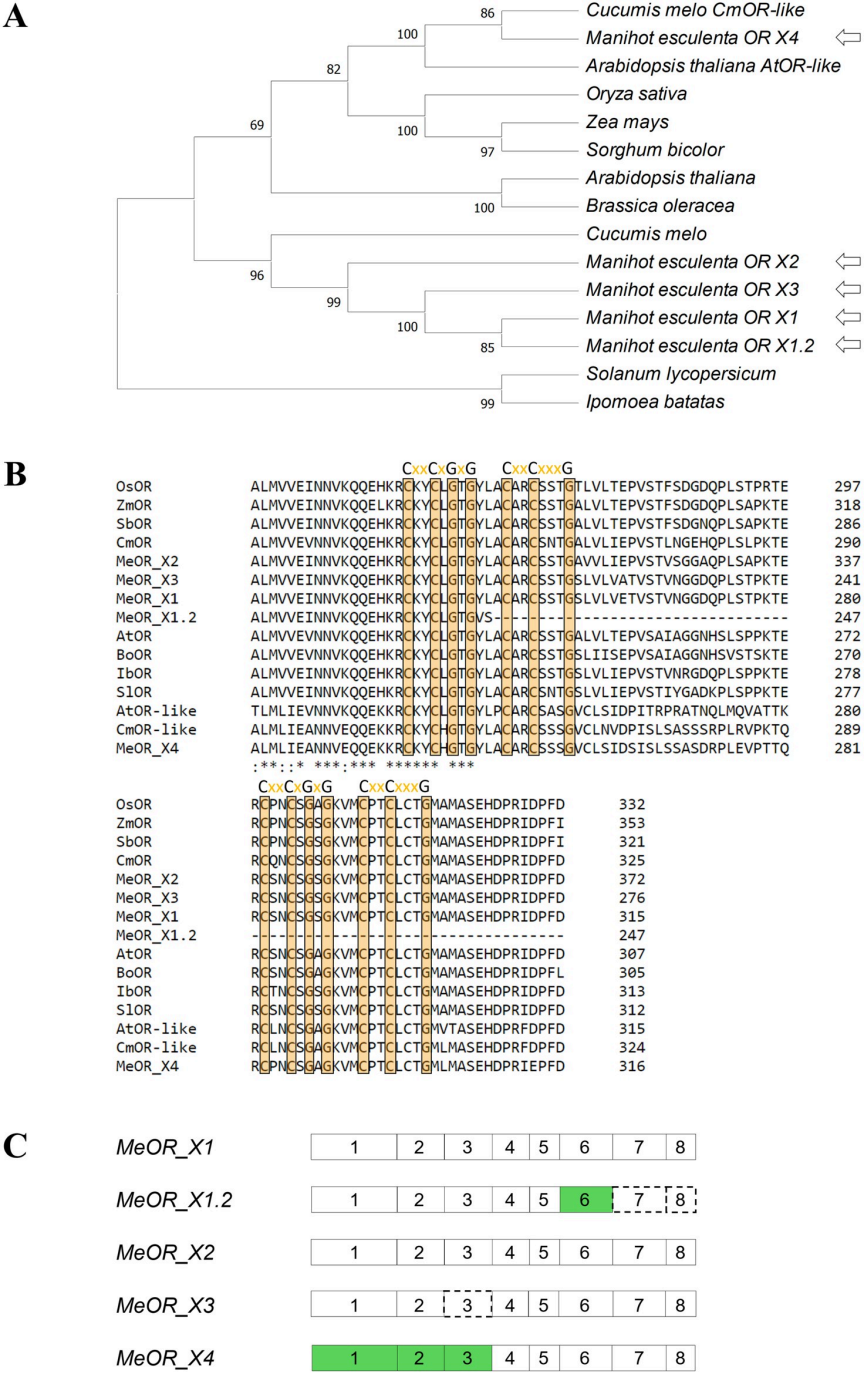
Characterization of cassava *OR* variants. (A) Phylogenetic tree of cassava OR predicted proteins and other OR proteins generated using a neighbor-joining method with MEGA-X. Bootstrap values obtained from 1000 iterations are shown at the beginning of each clade. (B) Clustal Omega alignment of the deduced amino acid sequences from the cassava *OR* variants and the genes of *Oryza sativa* (accession no. XP_015622925.1), *Zea mays* (accession no. ACN31016), *Sorghum bicolor* (accession no. XP_002452827), *Cucumis melo* (accession no. A0A0D3MU50.1), *Cucumis melo CmOR-like* (accession no. MELO3C024554), *Arabidopsis thaliana* (accession no. AT5G61670), *Arabidopsis thaliana AtOR-like* (accession no. AT5G06130), *Brassica oleracea* (accession no. A2T1U1.1), *Solanum lycopersicum* (accession no. NP_001315338.1), and *Ipomoea batatas* (accession no. APG21184.1). The four repeats of the highly conserved cys-rich domain are highlighted in orange and indicated above the alignment. (C) Schematic representation of cassava *OR* variant exons obtained from alignments conducted with the Splign tool. *MeOR*, cassava *OR* gene. Exon numbers presenting the same size among the cassava *OR* variants are represented in white boxes, exons with different sizes in gray, and exons missing with a broken line.

To investigate whether cassava OR presented the typical transmembrane regions of an OR protein, a prediction analysis using Phobious was carried out. All five OR putative variants in cassava contained the conserved transmembrane domains (S1 Fig). To evaluate closely related OR proteins among plants, a neighbor-joining phylogenetic tree of eight OR proteins from various plant species was built with MEGA-X (Fig 1A). Bootstrap values were obtained from 1000 iterations. MeOR_X1, MeOR_X1.2, MeOR_X2, and MeOR_X3 were closely related to and, apparently, they presented evolutionary similarities to CmOR as they formed a clade. Conversely, MeOR_X4 seemed to be evolutionarily closer to AtOR-like, CmOR-like and the cereal group of maize, sorghum, and rice. To study the presence of highly conserved regions, a Clustal Omega alignment was carried out (Fig 1B). Two highly conserved DnaJ-like cys-rich zinc-finger domains with two typical CxxCxGxG and CxxCxxxG motifs present in OR proteins [53] were found in all cassava variants, except for MeOR_X1.2, which presented just one. The full alignment can be found in S2 File. *MeOR_X1*.*2* presented the same first five exon sizes as *MeOR_X1* and *MeOR_X2* and differed slightly in the 3′ terminus of exon 6 (Fig 1C). Likewise, it missed exons 7 and 8 present in *MeOR_X1* producing a predicted smaller protein. All the variants except for *MeOR_X1*.*2* presented the same last five exon sizes, with a high sequence conservation (S2 File). *MeOR_X3* shared the same exon sizes as *MeOR_X1* and *MeOR_X2* but missed exon 3.

### Sequencing and SNP analysis

MeOR_X1 and MeOR_X2 were the putative proteins showing the highest identity compared to OR proteins from several plants (Table 1). Their potential implication in carotenoids accumulation was investigated. First, RNA was extracted from cassava roots using yellow genotype Y1. Then, RNA was reverse-transcribed and the genes *MeOR_X1* and *MeOR_2* were sequenced alongside *MePSY2* and *MePSY1* using the primers shown in S2 Table [27]. Subsequently, their sequences were compared with the sequences of those genes found in the cassava reference genome to search for potential SNPs. For this, an alignment using Clustal Omega was carried out. In total, 13 SNPs were found in the CDS regions, 7 synonymous and 6 non-synonymous (Table 2). Non-synonymous substitution T580A was found in *MeOR_X1*, G3A and A152T in *MeOR_X2*, A1154C and A1213C in *MePSY1*, and C572A in *MePSY2*, respectively. The first SNP in *MeOR_X2* (G3A) was positioned in the start codon ATG. The G3A substituted a methionine for an isoleucine residue generating MeOR_X2 G3A, with a new downstream start codon at position 172 which results in a protein which is N-terminally truncated by 57 amino acids. Interestingly, the new start codon coincided with the start of different OR proteins producing a protein with a MW of 34.4 kDa. Moreover, a cTP in MeOR_X2 was not predicted by ChloroP while a cTP was predicted for the protein encoded by MeOR_X2 G3A, similar to MeOR_X3 and MeOR_X4 (S3 Table). The second non-synonymous SNP in *MeOR_X2* (A152T) was located in the sequence area deleted by the modification in the start codon. A full alignment can be found in S3–S6 Files.

**Table 2:** Identification of SNPs in cassava *OR* and *PSY* variants.

Gene	SNP1	SNP2	SNP3	SNP4
*MeOR_X1*	TGT → AGT	-	-	-
	*Cys → Ser*			
*MeOR_X2*	ATG → ATA	CCT → CCA	CAC → CTC	AGA → AGG
	*Met → Ile*	Pro → Pro	*His → Leu*	Arg → Arg
*MePSY1*	GAC → GCC	TAC → TAT	AAG → CAG	AAA → AAG
	*Asp → Ala*	Tyr → Tyr	*Lys → Gln*	Lys → Lys
*MePSY2*	AAT → AAC	GCT → GAT	CTA → CTC	GCA → GCT
	Asn → Asn	*Ala → Asp*	Leu → Leu	Ala → Ala

*MeOR_X1*, *OR* cassava variant 1; *MeOR_X2*, *OR* cassava variant 2; *MePSY1*, cassava *phytoene synthase 1*; and *MePSY2*, cassava *phytoene synthase 2*. SNPs generating non-synonymous amino acids are highlighted in red and the amino acids are underlined and italicized.

To study whether the non-synonymous SNPs were exclusive from yellow lines, a differential diversity analysis in a broad germplasm collection consisting of 330 genotypes including white lines and colored lines was carried out (S1 File). Approximately 10 million high-quality SNP variants were retained after filtering. Among them, 12 out of the initially identified 13 loci of interest were found. Among the white genotypes, the reference alleles predominated for *MePSY1* and *MePSY2*. Conversely, in the yellow genotypes, the frequency of SNP1 (A1154C) of *MePSY1* and, remarkably, SNP2 (C572A) of *MePSY2* increased. On the contrary, the frequency of the alleles with the SNPs in the *MeOR* genes was high in both the white and colored genotypes, mainly in the homozygous form. The SNP3 (A1213C) could not be identified in the variation of the 330-sample population.

### Expression levels, protein abundance, and carotenoids content in cassava roots

**Fig 2:**
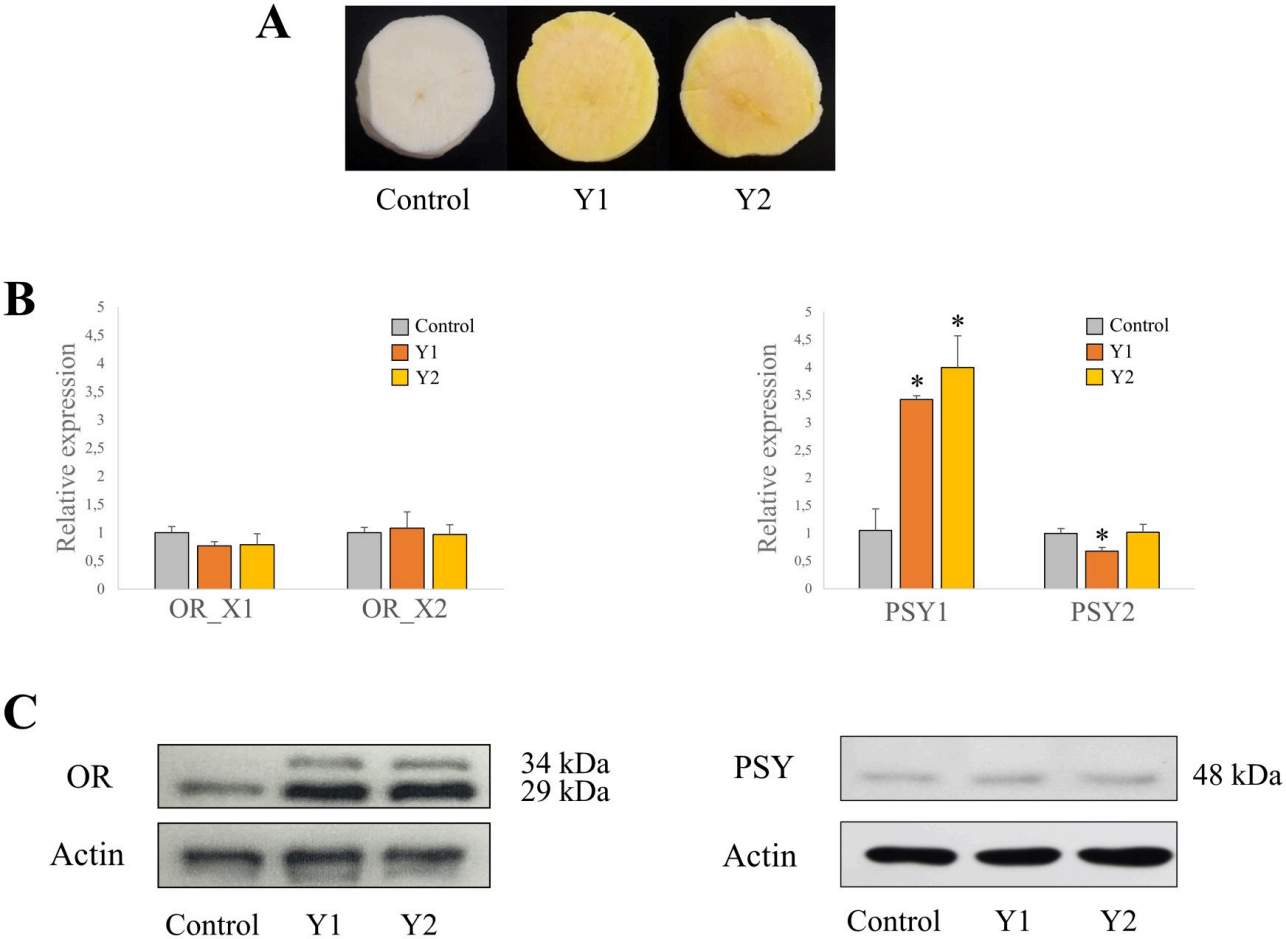
Expression and protein levels of OR and PSY in cassava roots. (A) Cross sections of white-fleshed genotype, and yellow-fleshed Y1, and Y2 cassava root genotypes. (B) Total carotenoid content of W, Y1 and Y2 genotypes measured by HPLC. (C) Relative expression levels of *MeOR_X1*, *MeOR_X2*, *MeOR_X4*, *MePSY1* and *MePSY2* in W, Y1, and Y2 genotypes by real-time qRT-PCR. (D) MeOR protein levels in the roots of W, Y1, and Y2 genotypes. Actin protein was used as a loading control. Values are the average ± SD of three biological replicates. *, Significant difference when compared to the white genotype, as determined by *t*-tests (*p*<0.05, *n* = 3). *MeOR_X1*, cassava variant 1; *MeOR_X2*, cassava variant 2; *MeOR_X4*, cassava variant 2; *MePSY1*, cassava *phytoene synthase 1*; *MePSY2*, cassava *phytoene synthase 2* [27].

To study the potential involvement of OR proteins in carotenoids accumulation, two yellow-fleshed cassava genotypes (Y1 and Y2, respectively) and one white-fleshed genotype 60444 (W) as a reference white line with poor carotenoids accumulation were used (Fig 2A). A higher carotenoids accumulation was confirmed in the yellow genotypes and β-carotene was the most abundant carotenoid (Fig 2B).

The expression levels of *MeOR_X1*, *MeOR_X2*, *MeOR_X4*, *MePSY1*, and *MePSY2* were analyzed by real-time qRT-PCR (Fig 2C). However, *MeOR_X1*.*2* and *MeOR_X3* were not amplified by real-time qRT-PCR using specific primer pairs, although endogenous controls (histidine and ubiquitin) were amplified as expected in the samples. The expression levels of *MeOR_X1*, *MeOR_X2* and *MeOR_X4* remained unchanged compared to the white roots. Likewise, the expression levels of *MePSY2* remained unchanged for genotype Y2, while in Y1 a significant decrease was observed. Conversely, *MePSY1* levels were up to 4-fold higher in both yellow genotypes compared to the white genotype. To study a possible alteration in β-carotene downstream metabolism, the expression levels of cassava *BCH1* and *9-cis epoxycarotenoid dioxygenase* (*NCED3*), the enzyme catalyzing ABA synthesis in cassava, were analyzed (S2 Fig). The expression levels of *BCH1* were lower in both yellow genotypes compared to the white one although significantly only in Y2. *NCED3* expression levels decreased up to 5- and 4-fold in the two yellow varieties, respectively.

Protein amount was analyzed by western blot using polyclonal antibodies (Fig 2D). For OR, one band with a MW of approximately 29 kDa was observed in both the white and the yellow genotypes, with significantly higher amounts in the yellow ones (S3 Fig). On the contrary, one band corresponding to a MW of around 34 kDa was observed exclusively in the yellow genotypes. No changes were observed between the white and yellow genotypes for PSY proteins, which present similar predicted sizes of approximately 48 kDa (Fig 2D).

### Cassava FEC transformation and protoplast visualization

**Fig 3:**
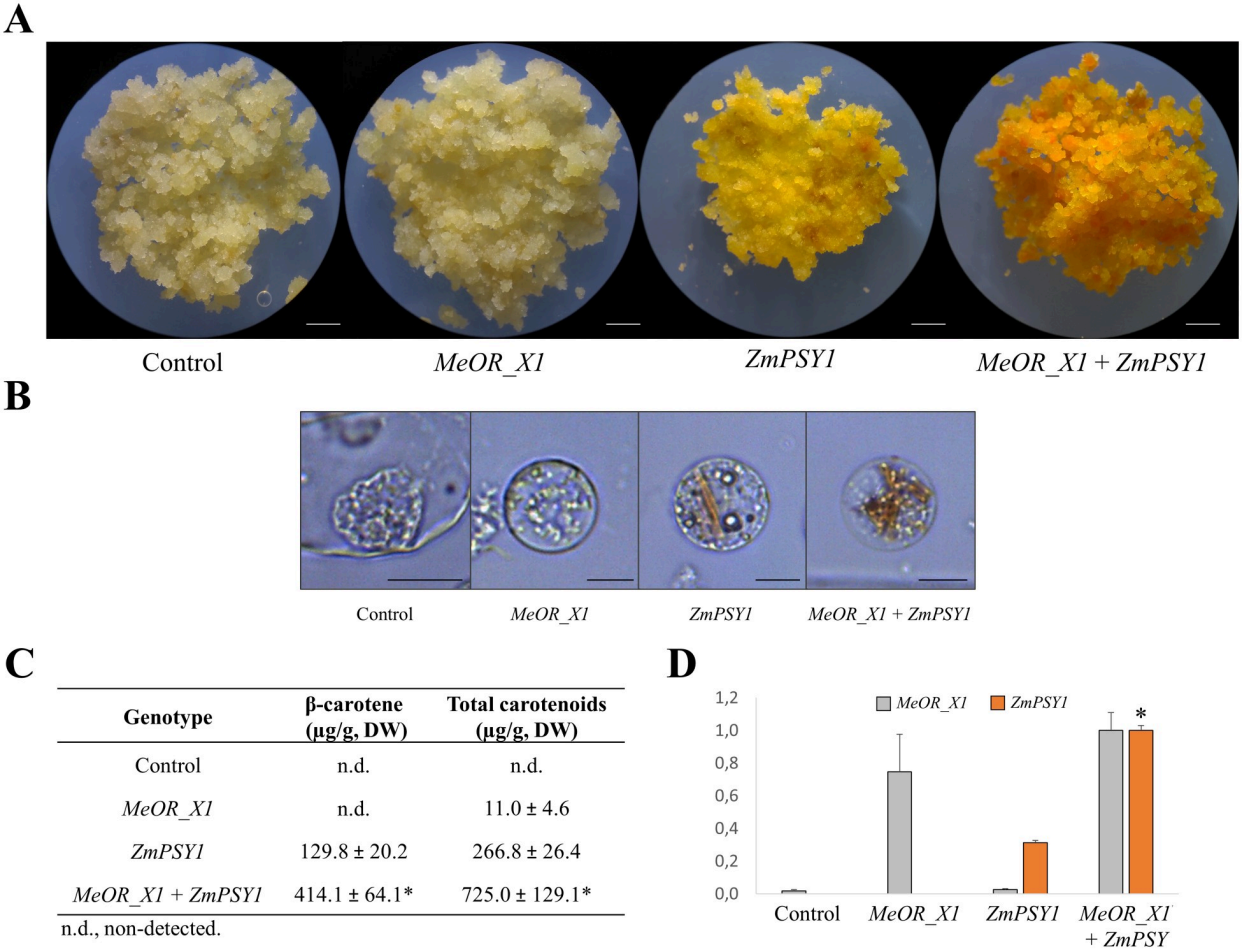
FEC and protoplasts of control, *MeOR_X1*, *ZmPSY1*, and *ZmPSY1* + *MeOR_X1* lines, carotenoid amounts and expression levels. (A) The FEC of Control, *MeOR_X1*, *ZmPSY1*, and *ZmPSY1* + *MeOR_X1* genotypes. (B) The protoplasts of Control, *MeOR_X1*, *ZmPSY1*, and *ZmPSY1* + *MeOR_X1* genotypes. (C) Total carotenoid content of Control, *MeOR_X1*, *ZmPSY1*, and *ZmPSY1* + *MeOR_X1* genotypes measured by HPLC. (D) Relative expression of *ZmPSY1* and *MeOR_X1* in Control, *MeOR_X1*, *ZmPSY1*, and *ZmPSY1* + *MeOR_X1* genotypes by real-time qRT-PCR. Values are the average ± SD of three biological replicates. *, Significant difference when compared to the callus transformed with *ZmPSY1*, as determined by *t*-tests (*p*<0.05, n = 3). Bars on the bottom right of picture indicate 2 mm for FEC and 15 μm for protoplast.

To investigate a direct implication of OR for the accumulation of carotenoids in cassava, the cassava *OR* variant *MeOR_X1* and the *ZmPSY1* were overexpressed in cassava FEC from genotype 60444 individually or combined in a tandem arrangement (*MeOR_X1*+*ZmPSY1)*. Maize PSY1 was used in expression and co-expression experiments as this PSY variant performed best in an array of PSYs from different taxa in rice endosperm and is frequently used in overexpression experiments [96, 101]. The transformation with just *MeOR_X1* produced the least intense orange color compared with those transformed with *ZmPSY1* and *ZmPSY1* + *MeOR_X1* (Fig 3A). The FEC co-transformed with *MeOR_X1*+*ZmPSY1* showed the most intense orange color. Freshly isolated protoplasts were used to visualize the formation of crystals. No carotenoid crystal formation was appreciated in the FEC protoplasts from the control and *MeOR_X1* lines. Conversely, crystals were formed in the *ZmPSY1* line. Noticeably, a massive accumulation of carotenoid crystals was observed in the co-transformed with *ZmPSY1* + *MeOR_X1* (Fig 3B). FEC transformed with *MeOR_X1* showed poor carotenoids accumulation, whereas the callus co-transformed with *MeOR_X1*+*ZmPSY1* presented the highest carotenoids accumulation, up to 3-4-fold higher compared to the FEC transformed with *ZmPSY1* alone (Fig 3C), β-carotene was the most abundant, but, interestingly, a higher level of other carotenoids was noted compared to the roots profile. While the expression levels of *MeOR_X1* were comparable in callus transformed with *MeOR_X1* alone and the callus co-transformed with *MeOR1_X1* and *ZmPSY1*, *ZmPSY1* levels were around 4-fold higher in callus co-transformed with *MeOR1_X1* and *ZmPSY1* compared with the FEC transformed with *ZmPSY1* alone (Fig 3D). Thus, the levels of *ZmPSY1* expression might explain most of the carotenoid content changes, while *MeOR1_X1* co-expression levels had only a minor contribution.

## Discussion

Vitamin A deficiency is a public health concern and is caused by a low dietary intake of this vitamin or its precursors [1, 102]. Biofortification of pVA in crops is a cost-effective strategy to maintain adequate vitamin A status in humans [103–106]. The high consumption of cassava in regions with micronutrient deficiencies makes it a suitable vehicle to supply pVA in the daily diet. However, the moderate content of pVA carotenoids in biofortified cassava can be decreased upon postharvest processing and storage conditions, thus can limit an adequate supply of pVA [19, 107]. The aim of this study was to investigate the potential of endogenous cassava OR to increase and stabilize carotenoids levels in this crop.

### Cassava contains four *OR* genes

Five putative cassava *OR* gene variants were identified in the cassava reference genome: three on chromosome 14, one on chromosome 6, and one on chromosome 9. The loci of these genes are different from the ones identified by Ovalle et al. [108], who defined two sites on chromosomes 2 and 7 that are related to carotenoids accumulation. Among the genes identified in our study, the cassava variant *MeOR_X1*.*2* seemed to be a splice variant of *MeOR_X1* since it shared the same loci at the same chromosome but misses the last two exons. *MeOR_X1*.*2* encoded a predicted protein of 27.4 kDa compared to the expected protein MW from MeOR_X1 of 34.4 kDa. In cauliflower, a retrotransposon insertion in *OR* produces the most abundant alternative spliced transcripts of 34.8 kDa, 32.6 kDa, and 29.6 kDa, respectively. However, no LTR retrotransposons were evidenced in cassava. OR proteins are characterized by harboring two transmembrane domains in the central region and a conserved cysteine-rich zinc-finger domain in the C-terminal region necessary for dimerization with four CxxCxGxG motifs [52, 56, 109]. All variants conserved the transmembrane domains. Conversely, *MeOR_X1*.*2* misses three CxxCxGxG motifs, which possibly alters the dimerization capacity of this variant and therefore its capacity to regulate PSY folding [52, 65]. Similarly, all *OR* variants except *MeOR_X1*.*2* presented the same sizes in the last five exons, along with highly conserved full sequences except for *MeOR_X4*. The phylogenetic analysis showed that variants *MeOR_X1*, *MeOR_X1*.*2*, *MeOR_X2*, and *MeOR_X3* form a highly conserved clade, which suggests at least one duplicated gene that might be a consequence of a whole-genome duplication [110]. *MeOR_X4* seems to be related to AtOR-like, CmOR-like and OR proteins from cereals. Nevertheless, a redundant function of OR variants could be expected as observed in other plants [56]. While transcripts of MeOR_X1.2 and MeOR_X3 were not detected in roots of any cassava variety included, expression levels of the other cassava OR variants showed no difference. Therefore, it remains to be determined whether MeOR_X1.2 and MeOR_X3 have distinct functions and are induced upon certain stimuli, similar to the situation for the PSY3 variants which are expressed upon arbuscular mycorrhizal formation [111].

The observation in the immunoblots of two bands corresponding to OR proteins of approximately 34 and 29 kDa in the yellow genotypes indicates a potential function of OR in the production or accumulation of carotenoids. The upper band of 34 kDa might correspond to the chloroplast imported variant MeOR_X2 G3A or to the variant MeOR_X4 with a predicted MW of 34.4 kDa (MeOR_X2 G3A) and 34.7 kDa (MeOR_X4). Likewise, the 34 kDa band may correspond to the non-imported MeOR_X1, with a molecular weight of 34.4 kDa. The cTP prediction for MeOR_X1 and its putative splice variant MeOR_X1.2 showed the same cleavage site score (CS-score) and the same predicted cTP length like the chloroplast-imported MeOR_X3 suggesting that these two OR variants could have been actually imported. The lower band of approximately 29 kDa may correspond to the imported MeOR_X3 with a MW of 29.8 kDa, or to the non-imported splice variant MeOR_X1.2 with a predicted MW of 27.2 kDa. Only small differences in the MW of the putative mature proteins would be expected after the excision of the predicted cTPs, thus further research such as a chloroplast import assay transiently expressing the OR variants to study their importation is encouraged [112].

### Contribution of nucleotide polymorphisms to carotenoid accumulation

In melon, an Arg to His “Golden SNP” is responsible for the accumulation of large amounts of carotenoids [53]. Overexpression of the Arabidopsis *OR* and sweet potato *OR* containing this SNP produces high amounts of carotenoids and can induce the biogenesis of membranous structures for carotenoids accumulation [67, 70]. However, the Arg to His SNP was not found in any of the cassava variants. SNPs in the *PSY* gene have been associated with a higher content of carotenoids in sweet potato, chili pepper (*Capsicum* spp.), and wheat [113–115]. In cassava, the content of carotenoids has been related to QTLs associated with a non-synonymous SNP2 of *PSY2* (C572A) [116–118]. The SNP2 (C572A) was identified in Y1, and almost all the genotypes from the differential diversity analysis that carried this SNP presented yellow color. However, approximately half of the colored genotypes did not carry this SNP. This observation confirmed previous research pointing to an additional regulatory mechanism for the biosynthesis and accumulation of carotenoids in cassava [28]. The first non-synonymous SNP found in *PSY1*, SNP1 (A1154C), seems not to be critical for carotenoids accumulation since only 19% of the genotypes with pigmentation harbored this SNP. Furthermore, although 63% of the genotypes that carried *PSY1* SNP1 presented pigmentation, these genotypes carried SNP2 also. Therefore, no specific function related to carotenoids accumulation could be attributed. Finally, the other non-synonymous SNP detected in *PSY1*, SNP3 (A1213C) was not found in any of the genotypes that fed the differential diversity analysis. This SNP might correspond to a random mutation on this locus of genotype Y1. In any case, this SNP seems not to be related to biosynthesis or accumulation of carotenoids.

Recent research with carrots has shown that a SNP in *OR* was associated with the accumulation of carotenoids [54]. We have identified one non-synonymous SNP in *MeOR_X1* and two in *MeOR_X2*. SNP1 in *MeOR_X2* (G3A) produces a change in the start codon decreasing the size of its CDS, which increases its identity with the other cassava OR variants and with OR from other plants. However, *MeOR* SNPs were detected in both white and yellow genotypes. Thus, they might not be particular for carotenoids production or stabilization.

### Posttranslational and mutual regulation of PSY and OR

Although OR is the major post-transcriptional regulator of PSY, the contrary can also occur and overexpression of *PSY* was shown to posttranslationally increase OR levels, resulting in mutual regulation [56]. In our study, despite the expression levels of *PSY2* remaining unchanged, surprisingly, an upregulation of 3- and 4-fold of *PSY1* was observed in Y1 and Y2, respectively. PSY protein content remained unchanged, but OR protein content was significantly higher, which may suggest a potential posttranslational regulatory mechanism of OR content by *PSY1*. This could be confirmed upon the overexpression of *PSY1*. Even though no increased accumulation of PSY has been observed in the yellow genotypes where OR accumulated, OR can stabilize active forms of PSY to continue contributing to high carotenoids formation [119]. Interaction studies are encouraged to confirm this. The upregulation of *PSY1* in roots with high carotenoid content could also respond to a high demand for PSY protein in case it presents low enzymatic activity. A similar mechanism was observed in tomato, where *PSY1*, which specifically expresses in fruits, is strongly expressed during ripening to compensate the very weak PSY1 enzymatic activity compared to the activity determined for the leaf-specific PSY2 [120]. According to our results, a direct relationship between *PSY1* and carotenoid accumulation could be considered. In conclusion, the enzymatic capacity of PSY proteins in cassava varieties with a high carotenoid content should be studied to understand the role of *PSY1* in the regulation of carotenoid production and accumulation.

### Implication of OR in carotenoids metabolism

The attenuation of β-carotene metabolism is one of the functions observed in OR [62]. In fact, a decrease in the expression of *BCH* was previously observed in roots of yellow cassava [121, 122]. Interestingly, we have observed a slight downregulation in the expression of *BCH1* in Y1 and a significant downregulation in expression in Y2. In cassava, an upregulation of *PSY1* responds to stress conditions, accompanied by an upregulation of *NCED* and greater ABA accumulation [25, 28]. However, the roots used in this study were not subjected to stress conditions and a significant decrease in *NCED3* expression level of up to 5- and 4-fold what was observed in genotypes Y1 and Y2, respectively. Interestingly, it was shown in a high carotenoid-producing tomato that a deficiency in ABA levels led to increased plastid number and a higher fruit lycopene content [123].

### Strategies to increase ∝-carotene amounts in cassava

The results of this study revealed that the regulatory mechanisms of carotenoids biosynthesis and accumulation in cassava seem to go beyond genes from the carotenoids biosynthesis pathway and OR SNPs or retrotransposons as it occurs in melon or cauliflower. Indeed, the attempts made so far to increase carotenoids content by overexpressing genes from the biosynthesis pathway could achieve only moderate levels even though bacterial genes were co-expressed [27, 29, 124]. Moreover, the levels achieved so far did not surpass those obtained by conventional breeding [24] and remain distant from those achieved in other plants [42, 125].

To study the potential implications for carotenoid accumulation, the variant *MeOR_X1* was overexpressed in cassava FEC tissue. One of the OR functions in plants is the stimulation of sink formation or crystallization for carotenoid accumulation and protection from degradation [50, 60, 70]. Therefore, it would be appropriate to transform a yellow cassava variety in which carotenoids presented a concentration high enough to eventually undergo accumulation and stabilization by the effect of OR. However, FEC transformation derived from yellow varieties has not yet been achieved and just a few genotypes such as the genotype 60444 was successfully transformed as this is a non-recalcitrant variety [94]. Thus, co-transformation with an exogenous *PSY* gene was necessary to study changes upon the presence of *MeOR_X1*. In fact, the FEC transformed with *MeOR_X1* presented only pale brownish pigmentation, β-carotene content was not detected, and the total carotenoid increase was low. This is expected considering that the effect of OR depends on PSY or the pathway activity and the white genotype 60444 has low biosynthesis capacity. Conversely, the FEC transformed with both *ZmPSY1* and *MeOR_X1* presented the highest carotenoid accumulation, with levels of β-carotene and total carotenoids more than 3-fold higher than in the FEC transformed with *ZmPSY1* only. Additionally, a stronger deposition of carotenoids in form of a massive crystal formation was observed in the protoplasts. One of the functions of OR proteins is the stimulation of crystals formation with the consequent stabilization of carotenoids [88, 126]. However, the expression levels of *ZmPSY1* in the callus co-transformed with *MeOR_X1* where almost 4-fold in comparison with the callus transformed with *ZmPSY1*, suggesting that cassava OR variant alone is not determinant for a higher accumulation and stabilization of carotenoids. A synergistic effect of the different OR variants might be necessary to enhance carotenoids accumulation as observed in other plants [52]. Besides its function to increase the carotenoid sink size, OR is thought to function as a holdase which prevents PSY from unfolding. Accordingly, seed-specific co-expression of the *OR*^*His*^ variant and *ZmPSY1* has a synergistic effect on Arabidopsis seed carotenoid content compared with lines expressing *ZmPSY1* solely [101]. However, the function of OR to increase the carotenoid sink size might be strongly dependent on the plastid type induced by the different OR variants as a similar synergistic effect was not observed in cassava FEC. Callus generate crystalline plastids in contrast to globular chromoplasts of Arabidopsis seeds which store lipophilic carotenoids in plastoglobuli. Moreover, maize PSY1 performed best among other putatively highly active PSY variants from other taxa (e.g. tomato, daffodil, pepper) in the endosperm of rice in developing Golden Rice 2 version which suggests that the unfolding protection of OR might strongly depend on PSY protein properties [96].

## Conclusions

In this study, we used different techniques that provided information about the mechanisms of action of OR in cassava. However, additional control mechanisms remain unclear and further research is necessary to address them. A higher accumulation of the protein OR and an upregulation of *PSY1* were observed in the yellow genotypes, which seems to indicate that both OR and the *PSY1* gene are involved in higher accumulation of carotenoids in yellow genotypes. Additional studies aimed at measuring the enzymatic capacity of PSY in cassava could generate more information about the role of this protein in the regulation of carotenoid accumulation. Furthermore, the overexpression independently and simultaneously of the *OR* variants that have not been tested in our study and their co-expression with the endogenous *PSY* genes might reveal new insights into the regulation of carotenoids production and accumulation in cassava.

## Supporting information

S1 Table Specific primers pairs for real time qPCR analysis. (PDF)

S2 Table Specific primer pairs for sequencing. (PDF)

S3 Table ChloroP predictions for chloroplasts transit peptides for cassava OR proteins. (PDF)

S1 Fig Transmembrane domains in cassava OR variants predicted with the Phobius tool. (TIF)

S2 Fig Relative expression levels of control, Y1 and Y2 lines in cassava roots by qRT-PCR. Values are the average ± SD of three biological replicates. *, Significant difference when compared to Control (*p* <0.05, *n* = 3). *NCED3*, *9-cis-epoxycarotenoid dioxygenase*; *BCH1*, *β-carotene hydroxylase*.
(TIF)

S3 Fig Western blot protein quantification. OR 29 kDa band. Actin-normalized protein levels relative to the white genotype (W) are shown above. Values are the average ± SD of three biological replicates. *, Significant difference when compared to the white genotype as determined by *t*-tests (*p* <0.05, *n* = 3). OR, Orange protein.
(TIF)

S1 File Differential diversity analysis. (XLSX)

S2 File Full length alignment of deduced amino acid sequences of cassava *OR* variants and *OR* genes from other plants. *Oryza sativa* (accession no. XP_015622925.1), *Zea mays* (accession no. ACN31016), *Sorghum bicolor* (accession no. XP_002452827), *Cucumis melo* (accession no. A0A0D3MU50.1), *Cucumis melo CmOR-like* (accession no. MELO3C024554), *Arabidopsis thaliana* (accession no. AT5G61670), *Arabidopsis thaliana AtOR-like* (accession no. AT5G06130), *Brassica oleracea* (accession no. A2T1U1.1), *Solanum lycopersicum* (accession no. NP_001315338.1) and *Ipomoea batatas* (accession no. APG21184.1). The alignment was carried out with Clustal Omega.
(PDF)

S3 File Alignment of full length of *MePSY1* CDS using Clustal Omega. Red arrows indicate the presence of a SNP.
(PDF)

S4 File Alignment of full length of *MePSY2* CDS using Clustal Omega. Red arrows indicate the presence of a SNP.
(PDF)

S5 File Alignment of full length of *MeOR_X1* CDS using Clustal Omega. Red arrows indicate the presence of a SNP.
(PDF)

S6 File Alignment of full length of *MeOR_X2* CDS using Clustal Omega. Red arrows indicate the presence of a SNP.
(PDF)

S1 Raw images (PDF)
