# Correction: Characterization of cassava ORANGE proteins and their capability to increase provitamin A carotenoids accumulation

**DOI:** 10.1371/journal.pone.0263168

**Published:** 2022-01-21

**Authors:** The PLOS ONE Staff

In the Discussion section, there is an error in the fifth sub-header. The correct sub-header is: Strategies to increase β-carotene amounts in cassava. The publisher apologizes for the error.
