# Supplementary material for: Characterization of cassava ORANGE proteins and their capability to increase provitamin A carotenoids accumulation
Source: PLoS One. 2022 Jan 7;17(1):e0262412. doi: 10.1371/journal.pone.0262412 (PMC8741059; doi:10.1371/journal.pone.0262412)
Supplement: S1 Table — (PDF) [file pone.0262412.s001.pdf]

S1 Table. Specific primers pairs for Real Time qPCR analysis

Primer	Sequence 5’ → 3'	Tm (°C)
UBI_F	AGCAGCTCGCATGTTCAGTGAGAA	60
UBI_R	TATAGACTGCTGGGCTGGTGCTTT	
HIS_F	ATTATGCAAGCGGGACAAAC	57
HIS_R	ACTCCACCGTACATCCTTGC	
MeOR_PSY1_F	GGATTAGGTAGTGGAAGCAA	58
MeOR_PSY1_R	CTGTTTTCGCATCCAGATTC	
MeOR_PSY2_F	ACTTGCATAGGTAGCGGAC	58
MeOR_PSY2_R	TGGCTATTTCTCCTGCGTGA	
MeOR_OR_X1_F	ATGCTTGTCCGGTTCTCACG	57
MeOR_OR_X1_R	GCGGCGTTTTTATCAGTGGA	
MeOR_OR_X2_F	ACTGGAATGGCTATGGCAAGT	57
MeOR_OR_X2_R	TGCTATGCCACTCCAGAGAC	
MeOR_OR_X4_F	TCTCTGCATGAGCTCACACA	58
MeOR_OR_X4_R	AGGAGGAGCGGTTGGATAGT	
ZmPSY_F	CAGGCCGCATTGCTCAAAC	58
ZmPSY_R	CACCACACATAGATGGCCCA	
NCED_F	GAAATGATTCGCGGTGGCTC	58
NCED_R	TCTTGGAAGCGTCATTGGCT	
BCH_F	TGGCACGTTGGCAATGG	58
BCH_R	TTGTACACGATGGTCTTGTTCACA	
