# Supplementary material for: Characterization of cassava ORANGE proteins and their capability to increase provitamin A carotenoids accumulation
Source: PLoS One. 2022 Jan 7;17(1):e0262412. doi: 10.1371/journal.pone.0262412 (PMC8741059; doi:10.1371/journal.pone.0262412)
Supplement: S2 Table — (PDF) [file pone.0262412.s002.pdf]

S2 Table. Specific primer pairs for sequencing

Gene	Sequence 5' → 3'
PSY1	TGCTGTTCAATCCCCTTGGG (F1)
	TTGCTTTCACCTTTTTCATCTGT (R1)
	GGATTAGGTAGTGGAAGCAA (F2)
	GATGGGAGGCAAGGTTGGAA (F3)
PSY2	AGCTGAGCTTCTTTCGCTGT (F1)
	ATTGATGTTCATGGTTTGCACA (R1)
	ACTTGCATAGGTAGCGGAC (F2)
	TGGCTATTTCTCCTGCGTGA (R2)
OR_X1	TCGTTCTTCACCTGGCTTCA (F1)
	CAGAGAGGGAGGGGGTCATC (R1)
	ATGCTTGTCCGGTTCTCACG (F2)
	GCGGCGTTTTTATCAGTGGA (R2)
OR_X2	CCAACAGCCAGTGTCTTCCT (F1)
	TGGAAATCTATGGCACCGAGG (R1)
	CTGTCTGGTCCTCACAGCTT (F2)
	GGCGGCGGTTTTATCAGTTG (R2)
