# Supplementary material for: Characterization of cassava ORANGE proteins and their capability to increase provitamin A carotenoids accumulation
Source: PLoS One. 2022 Jan 7;17(1):e0262412. doi: 10.1371/journal.pone.0262412 (PMC8741059; doi:10.1371/journal.pone.0262412)
Supplement: S3 Table — (PDF) [file pone.0262412.s003.pdf]

S3 Table. ChloroP predictions for chloroplasts transit peptides for cassava OR proteins.

Variant name	Lenght	Score	Chloroplast Transit Peptide (cTP)	Cleavage site score (CS-score)	cTP lenght
MeOR_X1	315	0.495	-	3.247	46
MeOR_X1.2	247	0.495	-	3.247	46
MeOR_X2	372	0.454	-	0.264	63
MeOR_X2 G3A	315	0.532	Yes	3.776	46
MeOR_X3	276	0.510	Yes	3.247	46
MeOR_X4	316	0.576	Yes	0.231	55
