# Supplementary figures and images for: Characterization of cassava ORANGE proteins and their capability to increase provitamin A carotenoids accumulation
Source: PLoS One. 2022 Jan 7;17(1):e0262412. doi: 10.1371/journal.pone.0262412 (PMC8741059; doi:10.1371/journal.pone.0262412)

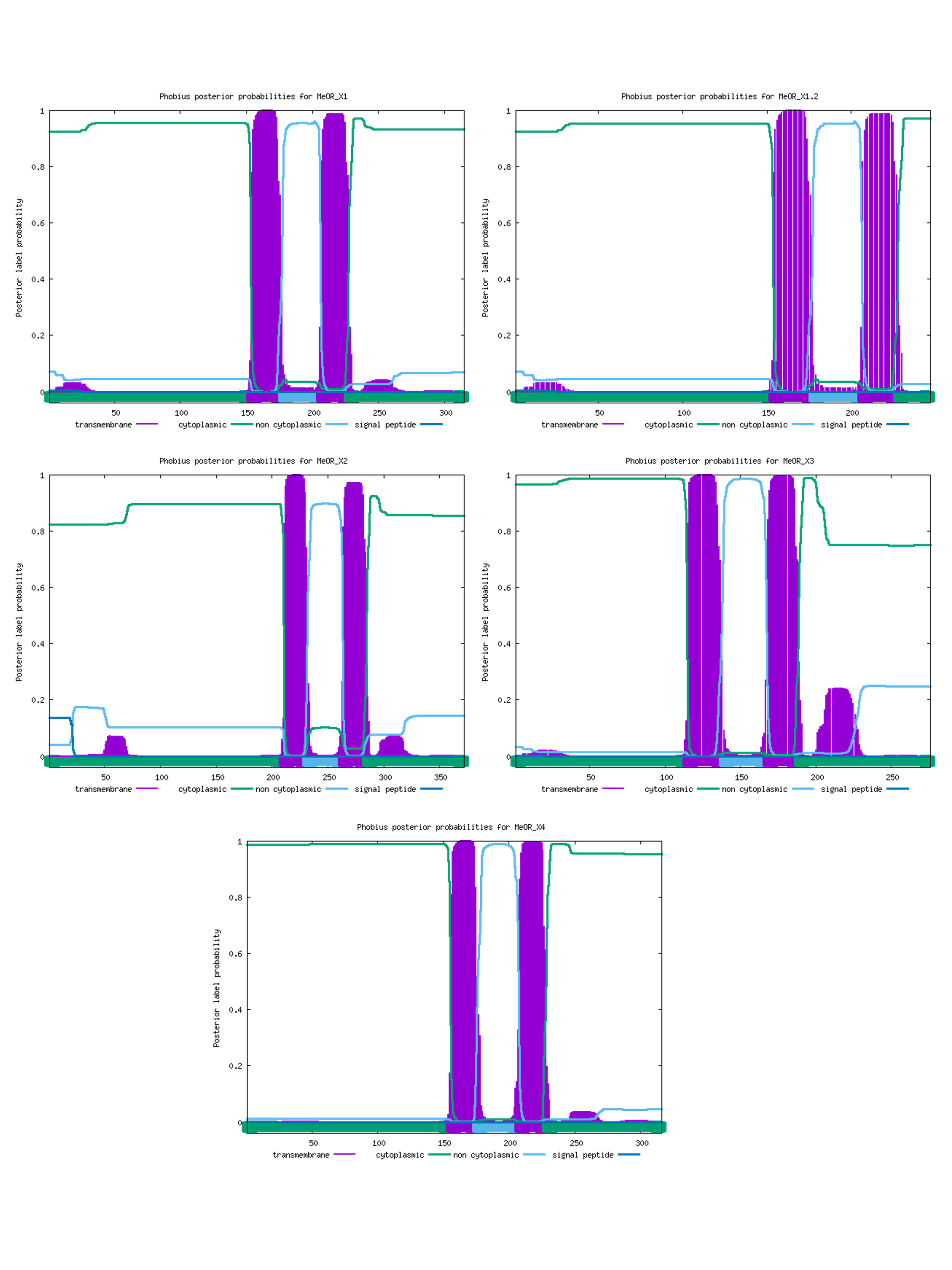

Supplement: S1 Fig — (TIF) [file pone.0262412.s004.tif]

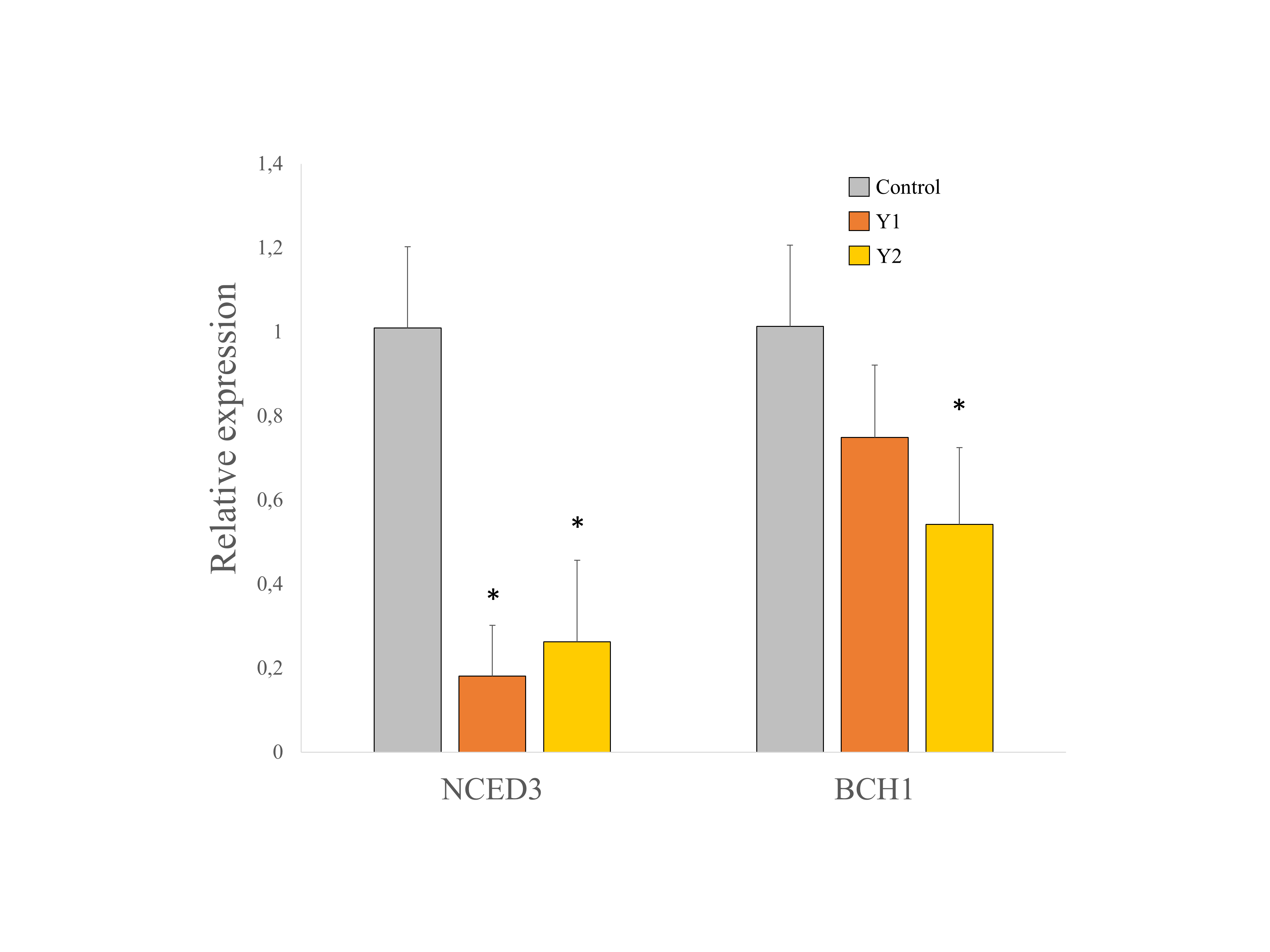

Supplement: S2 Fig — Values are the average ± SD of three biological replicates. *, Significant difference when compared to Control (p <0.05, n = 3). NCED3, 9-cis-epoxycarotenoid dioxygenase; BCH1, β-carotene hydroxylase. (TIF) [file pone.0262412.s005.tif]

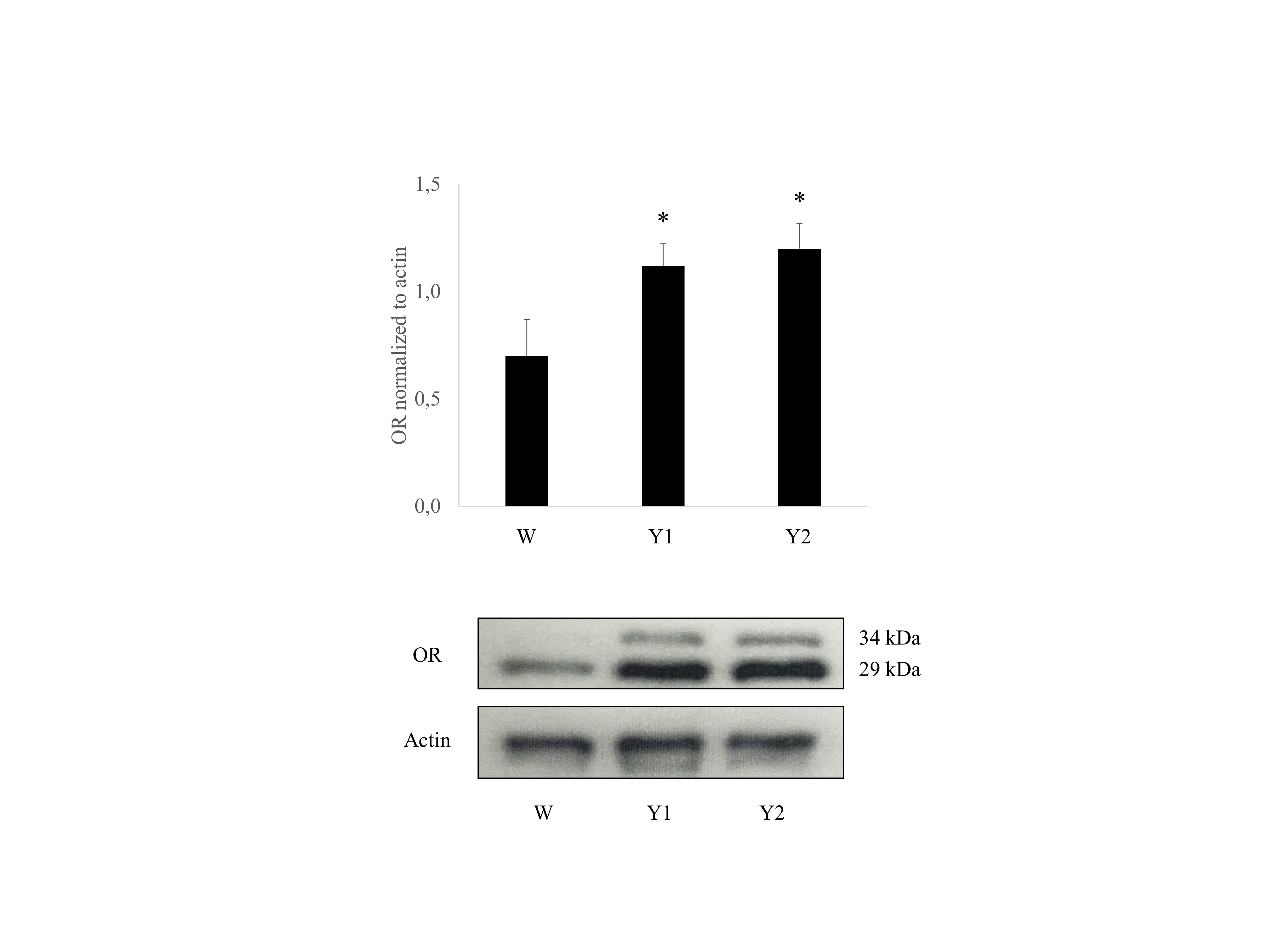

Supplement: S3 Fig — OR 29 kDa band. Actin-normalized protein levels relative to the white genotype (W) are shown above. Values are the average ± SD of three biological replicates. *, Significant difference when compared to the white genotype as determined by t-tests (p <0.05, n = 3). OR, Orange protein. (TIF) [file pone.0262412.s006.tif]

PSY Western blots scans  
(CIAT)

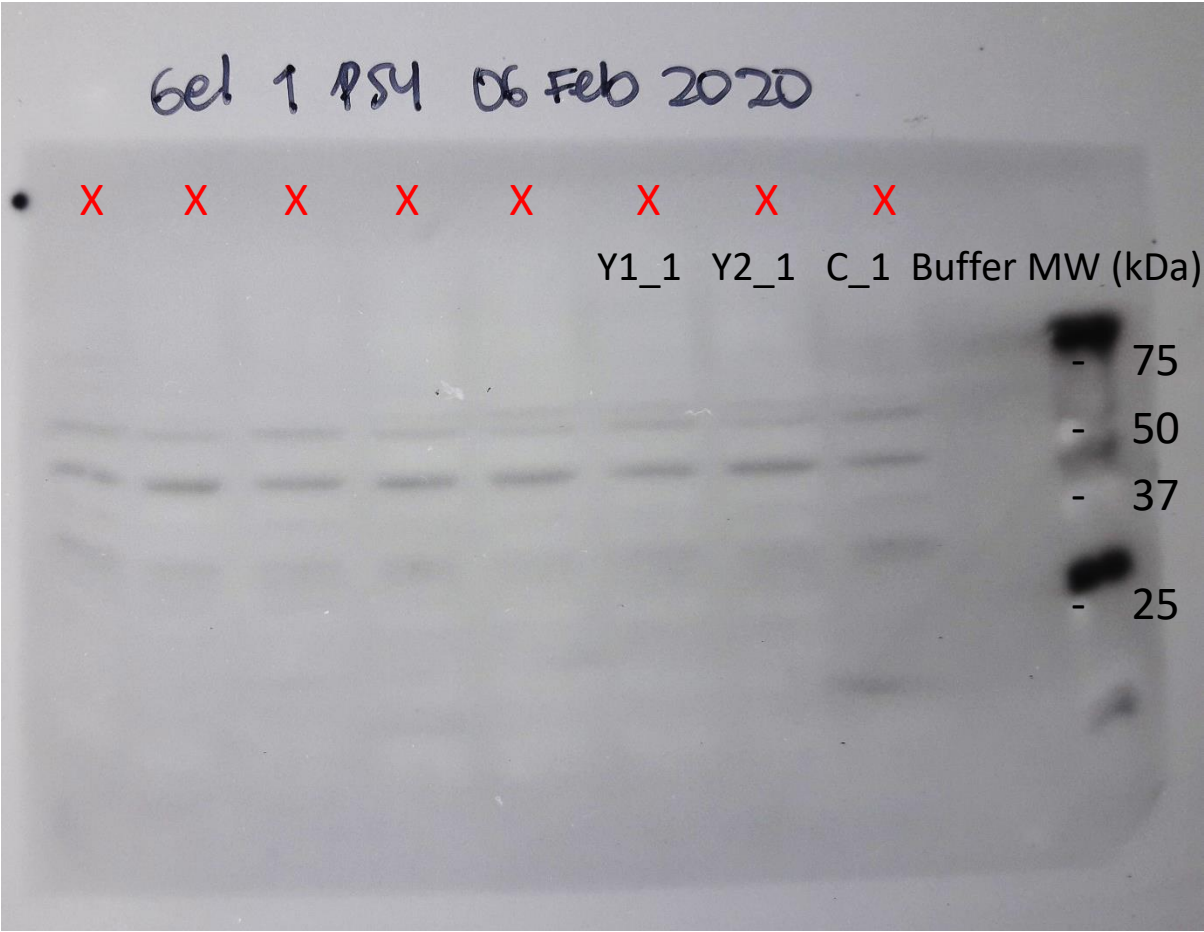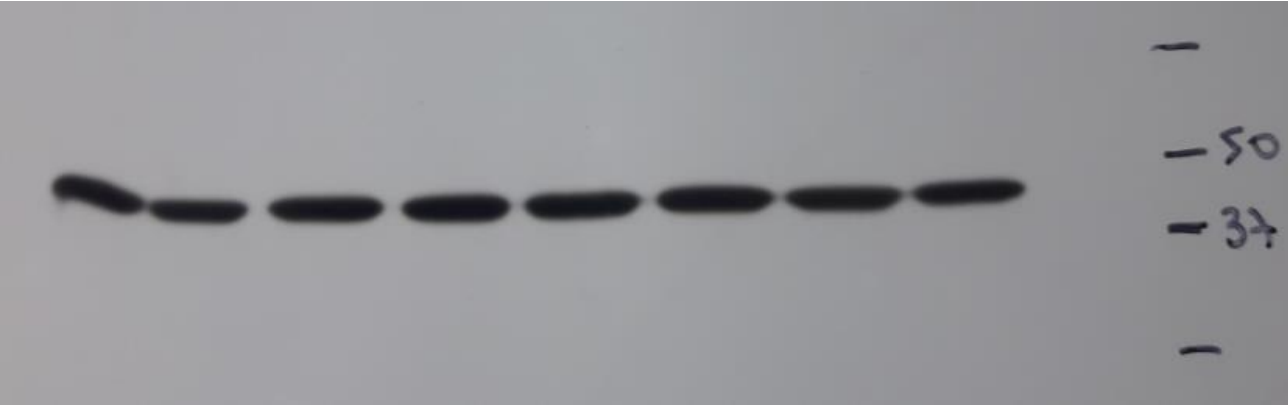

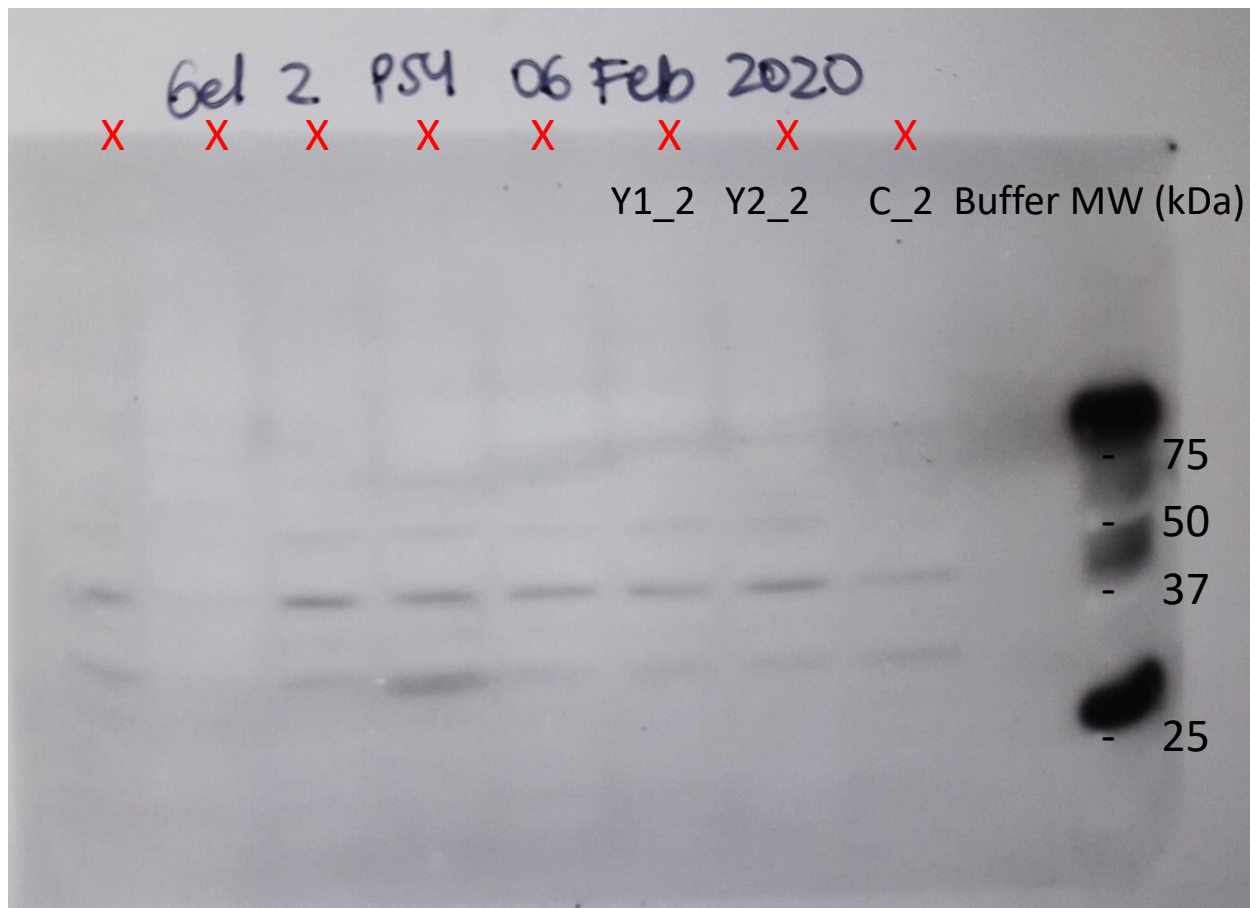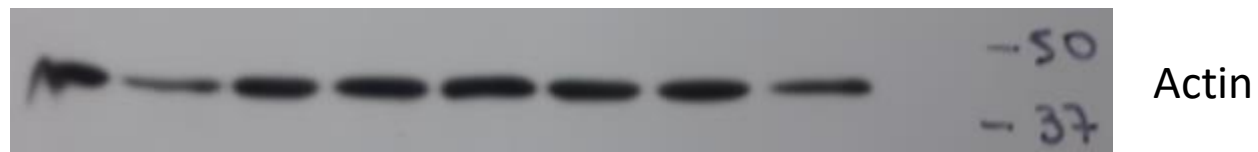

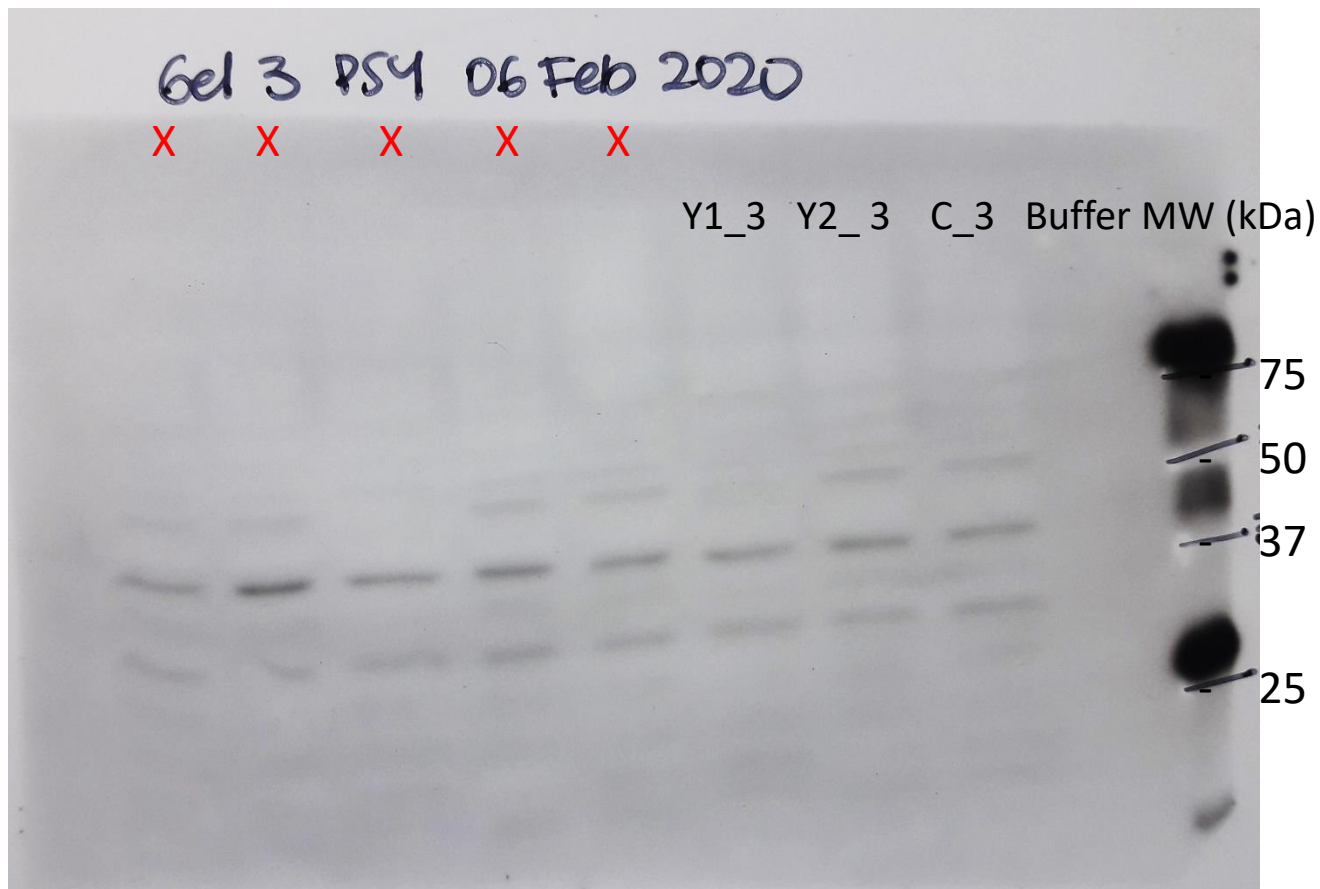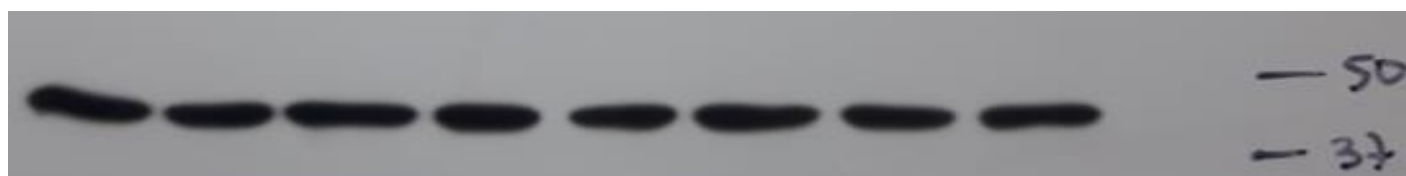

Actin

## OR Western blots scans (Cornell University)

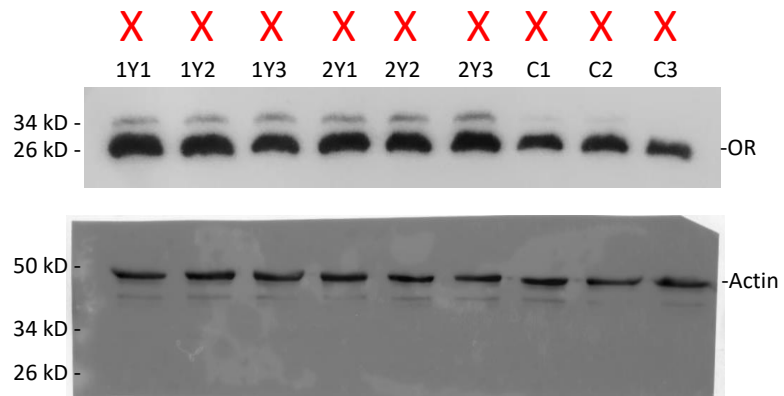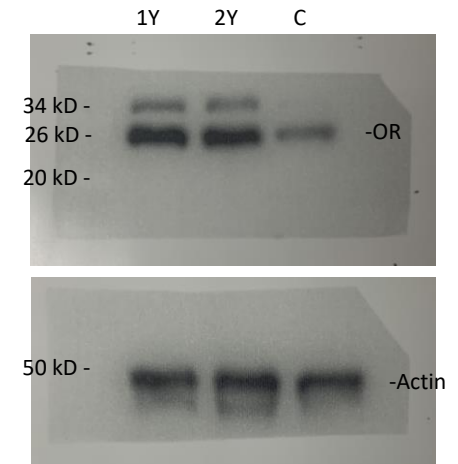

Supplement: S1 Raw images — (PDF) [file pone.0262412.s013.pdf]
