# Supplementary material for: Characterization of cassava ORANGE proteins and their capability to increase provitamin A carotenoids accumulation
Source: PLoS One. 2022 Jan 7;17(1):e0262412. doi: 10.1371/journal.pone.0262412 (PMC8741059; doi:10.1371/journal.pone.0262412)
Supplement: S2 File — Oryza sativa (accession no. XP_015622925.1), Zea mays (accession no. ACN31016), Sorghum bicolor (accession no. XP_002452827), Cucumis melo (accession no. A0A0D3MU50.1), Cucumis melo CmOR-like (accession no. MELO3C024554), Arabidopsis thaliana (accession no. AT5G61670), Arabidopsis thaliana AtOR-like (accession no. AT5G06130), Brassica oleracea (accession no. A2T1U1.1), Solanum lycopersicum (accession no. NP_001315338.1) and Ipomoea batatas (accession no. APG21184.1). The alignment was carried out with Clustal Omega. (PDF) [file pone.0262412.s008.pdf]

CLUSTAL O(1.2.4) multiple sequence alignment

```
SlOR            ---------------------------------------------------------MVC 3
OsOR            ---------------------------------------------------------MLC 3
ZmOR            ---------------------------------------------------------MLC 3
SbOR            ---------------------------------------------------------MLC 3
CmOR            ------------------------------------------------------------ 0
AtOR            ---------------------------------------------------------MSS 3
BoOR            ---------------------------------------------------------MSC 3
IbOR            ---------------------------------------------------------MVY 3
MeOR_X2         MLHLQLLCFSCLYYPPSPDSDKQLQIFLRLRRNKKFFVSILLLQQYETETHRLGVSIMGS 60
MeOR_X3         ---------------------------------------------------------MGS 3
MeOR_X1         ---------------------------------------------------------MGS 3
MeOR_X1.2       ---------------------------------------------------------MGS 3
AtOR-like       ---------------------------------------------------------MTC 3
CmOR-like       ------------------------------------------------------------ 0
MeOR_X4         ---------------------------------------------------------MT- 2

SlOR            AGRILYLSCSTTP------------FSPST------------------------------ 21
OsOR            SARMLACSGLGGPGGR---------LRPSPRP-----------G------AYADRLR--- 34
ZmOR            SGRMLACNGVLPG--R---------LR---LP-----------R------GDAYHLR--- 29
SbOR            SGRMLACNGLLPG--R---------LR---LP-----------R------ADAYRLR--- 29
CmOR            MDRVLVASY--PINHL---------IRPHSFRIDYCWSTCFTSR------LN--SGKERQ 41
AtOR            LGRILSVSY--PPD-------------------PYTWRFS------------QYKLSSSL 30
BoOR            LGRILSVSY--PPD-------------------PYGSRLS------------VSKLS-SP 29
IbOR            SGRILSLSS--STT-------------------PFHLST-SPFH------SSRYHL-HG- 33
MeOR_X2         LSRVLAVSY--PHK-------------------PLCLSGPHSLH------HSNYRF-VDV 92
MeOR_X3         LGRVLAVLY--PNK-------------------LLSLSGSHGLH------HLNCRF-KGF 35
MeOR_X1         LGRVLAVLY--PNK-------------------LLCLSGSHGLH------HLNCRF-KGF 35
MeOR_X1.2       LGRVLAVLY--PNK-------------------LLCLSGSHGLH------HLNCRF-KGF 35
AtOR-like       FS------SATPHRHHLLLSSPSTSKSLLRFPSSYLKPSPSL------------------ 39
CmOR-like       ------------------MSLPSTFAPSSHFPLPISHFKPSRHCRASIFLSIDYGCCKSL 42
MeOR_X4         ----------------DAFSLCSHHRLLSPFPISSSKSKPHVFSNRLSLL---------- 36

SlOR            ------------------------------------------------------------ 21
OsOR            ----PPLPARRWRVASSAAASGGSPDLPSSSSSSSPPPTPAAASFGSGDEQAAGSPGFCI 90
ZmOR            ----PPALARRWRVVASAAASGGSPDLPSSSSSPP------NPPFGAGDDQTAASPGFCI 79
SbOR            ----PPALARRWSVAASAAASGGSSDLPSSSSSPP------TPPFGVGDDQAAASPGFCI 79
CmOR            ----KLSSRWRWRSMASDSTDSSS-----SSS--F-------APSVESDPSDKTSASFCI 83
AtOR            ----GRNRRLRWRFTALDP----E-----SSS--L-------DS---ESSADKFASGFCI 65
BoOR            ----GRNRRLRWRFTALDS----D-----SSS--L-------DS---DS-SDKFAAGFCI 63
IbOR            ----RLKSRVRLRPMAADA----D-----SSS--F-------SSSVDTESPDKNAAGFCI 71
MeOR_X2         ----NRKSSSKWRSMASEL----E-----SSS--F-------SPSIDSDSTDKTAAGFCI 130
MeOR_X3         ----DRKLSSKWRSMASEF----E-----SSS--F-------APSGDSDSTDNNAAGFCI 73
MeOR_X1         ----DRKLSSKWRSMASEF----E-----SSS--F-------APSVDSDSTDKNAAGFCI 73
MeOR_X1.2       ----DRKLSSKWRSMASEF----E-----SSS--F-------APSVDSDSTDKNAAGFCI 73
AtOR-like       ------LFHGSSRSLLSC---------------------SDGSNNRPPPSGDTVPNNFCI 72
CmOR-like       YASSRNTSRRSLRICASS---------------------SDGASASVPSDSDNTPSNFCI 81
MeOR_X4         ----SNRSSSLFRILCSS---------------------PINSSD-SAPSGDRLSSNFCI 70

SlOR            ------------------------------------------------------------ 21
OsOR            IEGPETVQDFEKLDLQEIQDNIRSRRNKIFLHMEE------------------------- 125
ZmOR            IEGPETVQDFAKLDLQEIQDNIRSRRNKIFLHMEEMVWGILVQLNGSQSKIKQNNRLLNL 139
SbOR            IEGPETVQDFAKLDLQEIQDNIRSRRNKIFLHMEE------------------------- 114
CmOR            IEGPETVQDFAKMELQEIQENIRSHRNKIFLHMEE------------------------- 118
AtOR            IEGPETVQDFAKMQLQEIQDNIRSRRNKIFLHMEE------------------------- 100
BoOR            IEGPETVQDFAKMQLQEIQDNIRSRRNKIFLHMEE------------------------- 98
IbOR            IEGPETVQDFAQMELKEIQDNIRSRRNKIFLHMEE------------------------- 106
MeOR_X2         IEGPETVQDLSKMELQEIRDNIRSRRNKIFLHMEE------------------------- 165
```

```
MeOR_X3        IEGPETVQDFAKMELQEIRDNIRSRRNKIFLQMEE------------------------- 108
MeOR_X1        IEGPETVQDFAKMELQEIRDNIRSRRNKIFLQMEE------------------------- 108
MeOR_X1.2      IEGPETVQDFAKMELQEIRDNIRSRRNKIFLQMEE------------------------- 108
AtOR-like      IEGSETVQDFVQMQLQEIQDNIRSRRNKIFLLMEE------------------------- 107
CmOR-like      IEGPETVQDFVQMQFQEIQDNIRSRRNKIFLLMEE------------------------- 116
MeOR_X4        IEGPETVQDFVQMQLQEIQDNIRSRRNKIFLLMEE------------------------- 105


SlOR           ------------------------------------------------------------ 21
OsOR           -------IRRLRIQQRIKNVELGISV----DVPEGELPDFPSFIPFLPPLSAANLKIYYA 174
ZmOR           QCLDLSMIRRLRIQQRIKNVELGISD----EERDHELPDFPSFIPFLPPLSAANLKVYYA 195
SbOR           -------IRRLRIQQRIKNVELGISD----EESDRELPDFPSFIPFLPPLSAANLKVYYA 163
CmOR           -------VRRLRIQQRIKNAELGISK----EERENELPNFPSFIPFLPPLSSENLKLYYV 167
AtOR           -------VRRLRIQQRIKNTELGIIN----EEQEHELPNFPSFIPFLPPLTAANLKVYYA 149
BoOR           -------VRRLRIQQRIRNTELGIID----EEQEHELPNFPSFIPFLPPLTAANLRVYYA 147
IbOR           -------VRRLRIQQRIKNAELGILN----EKQENELPNFPSFIPFLPPLTSANLKQYYA 155
MeOR_X2        -------VRRLRIQQRIKSAELGILK----ETQENELPNFPSFIPFLPPLSAENLKLYYA 214
MeOR_X3        --------------------------------------------------SAENLKLYYA 118
MeOR_X1        -------VRRLRIQQRIKSAELGILK----EDHEHELPDFPSFIPFLPPLSAENLKLYYA 157
MeOR_X1.2      -------VRRLRIQQRIKSAELGILK----EDHEHELPDFPSFIPFLPPLSAENLKLYYA 157
AtOR-like      -------VRRLRVQQRIKSVKAINEDS---ELEATEMPEITSSIPFLPNVTPKTLKQLYS 157
CmOR-like      -------VRRLRIQQRLKNLKAIDEND---NEEAYEMPEIPSSIPFLPHVTPKTLKQQYL 166
MeOR_X4        -------VRRLRVQQRIKSVKVIDENVEEEEEEADEMPEMPSSIPFLPRVTPKTLKQLYL 158


SlOR           ------------------------------------------------------------ 21
OsOR           TCFTLIAGIMVFGGFLAPILELKLGVGGTSYADFIRSVHLPMQLSQVDPIVASFSGGAVG 234
ZmOR           TCFTLIAGIMVFGGFLAPILELKLGVGGTSYEDFIRSVHLPMQLSQVDPIVASFSGGAVG 255
SbOR           TCFALIASIMVFGGLLAPILELKLGLGGTSYEDFIRSVHLPMQLSEVDPIVASFSGGAVG 223
CmOR           TCYSLIAGIILFGGLLAPTLELKLGLGGTSYEDFIRSVHLPMQLSQVDPIVASFSGGAVG 227
AtOR           TCFSLIAGIILFGGLLAPTLELKLGIGGTSYADFIQSLHLPMQLSQVDPIVASFSGGAVG 209
BoOR           TCFSLIAGIILFGGLLAPTLELKLGIGGTSYKDFIQSLHLPMQLSQVDPIVASFSGGAVG 207
IbOR           TCFSLIAGVMLFGGLLAPTLELKLGLGGTSYADFIRSMHLPMQLSDVDPIVASFSGGAVG 215
MeOR_X2        TCFSLIAGIIIFGGLLAPTLELKLGLGGTSYEDFIRSVHLPMQLSQVDPIVASFSGGAVG 274
MeOR_X3        TCFSLIAGIIIFGGLLAPILEIKLGLGGTSYADFIRSVHLPMQLSQVDPIVASFSGGAVG 178
MeOR_X1        TCFSLIAGIIIFGGLLAPILEIKLGLGGTSYADFIRCVHLPMQLSQVDPIVASFSGGAVG 217
MeOR_X1.2      TCFSLIAGIIIFGGLLAPILEIKLGLGGTSYADFIRCVHLPMQLSQVDPIVASFSGGAVG 217
AtOR-like      TSVALISGIIFFGGLIAPNLELKVGLGGTSYEDFIRSLHLPLQLSQVDPIVASFSGGAVG 217
CmOR-like      TSLSVIWGIIVFGGFIAPTLELKLGLGGTSYEDFIRNMHLPMQLSQVDPIVASFSGGAVG 226
MeOR_X4        TSLSFISGIIAFGGLIAPTLELKLGLGGTSYEDFIRSMHLPMQLSQVDPIVASFSGGAVG 218


SlOR           ------------------------------------------------------------ 21
OsOR           VISALMVVEINNVKQQEHKRCKYCLGTGYLACARCSSTGTLVLTEPVSTFSDGDQPLSTP 294
ZmOR           VISALMVVEINNVKQQELKRCKYCLGTGYLACARCSSTGALVLTEPVSTFSDGDQPLSAP 315
SbOR           VISALMVVEINNVKQQEHKRCKYCLGTGYLACARCSSTGALVLTEPVSTFSDGNQPLSAP 283
CmOR           VISALMVVEVNNVKQQEHKRCKYCLGTGYLACARCSNTGALVLIEPVSTLNGEHQPLSLP 287
AtOR           VISALMVVEVNNVKQQEHKRCKYCLGTGYLACARCSSTGALVLTEPVSAIAGGNHSLSPP 269
BoOR           VISALMVVEVNNVKQQEHKRCKYCLGTGYLACARCSSTGSLIISEPVSAIAGGNHSVSTS 267
IbOR           VISALMVVEINNVKQQEHKRCKYCLGTGYLACARCSSTGSLVLIEPVSTVNRGDQPLSPP 275
MeOR_X2        VISALMVVEINNVKQQEHKRCKYCLGTGYLACARCSSTGAVVLIEPVSTVSGGAQPLSAP 334
MeOR_X3        VISALMVVEINNVKQQEHKRCKYCLGTGYLACARCSSTGSLVLVATVSTVNGGDQPLSTP 238
MeOR_X1        VISALMVVEINNVKQQEHKRCKYCLGTGYLACARCSSTGSLVLVETVSTVNGGDQPLSTP 277
MeOR_X1.2      VISALMVVEINNVKQQEHKRCKYCLGTGVS------------------------------ 247
AtOR-like      VISTLMLIEVNNVKQQEKKRCKYCLGTGYLPCARCSASGVCLSIDPITRPRATNQLMQVA 277
CmOR-like      VISALMLIEANNVEQQEKKRCKYCHGTGYLACARCSSSGVCLNVDPISLSASSSRPLRVP 286
MeOR_X4        VISALMLIEANNVEQQEKKRCKYCHGTGYLACARCSSSGVCLSIDSISLSSASDRPLEVP 278


SlOR           ----------------------------------SAF-       24
OsOR           RTERCPNCSGAGKVMCPTCLCTGMAMASEHDPRIDPFD       332
ZmOR           KTERCPNCSGSGKVMCPTCLCTGMAMASEHDPRIDPFI       353
SbOR           KTERCPNCSGSGKVMCPTCLCTGMAMASEHDPRIDPFI       321
```

CmOR	KTERCQNCSGSGKVMCPTCLCTGMAMASEHDPRIDPFD	325
AtOR	KTERCSNCSGAGKVMCPTCLCTGMAMASEHDPRIDPFD	307
BoOR	KTERCSNCSGAGKVMCPTCLCTGMAMASEHDPRIDPFL	305
IbOR	KTERCTNCSGSGKVMCPTCLCTGMAMASEHDPRIDPFD	313
MeOR_X2	KTERCSNCSGSGKVMCPTCLCTGMAMASEHDPRIDPFD	372
MeOR_X3	KTERCSNCSGSGKVMCPTCLCTGMAMASEHDPRIDPFD	276
MeOR_X1	KTERCSNCSGSGKVMCPTCLCTGMAMASEHDPRIDPFD	315
MeOR_X1.2	--------------------------------------	247
AtOR-like	TTKRCLNCSGAGKVMCPTCLCTGMVTASEHDPRFDPFD	315
CmOR-like	KTQRCLNCSGAGKVMCPTCLCTGMLMASEHDPRFDPFD	324
MeOR_X4	TTQRCPNCSGAGKVMCPTCLCTGMLMASEHDPRIEPFD	316
