# Supplementary material for: Characterization of cassava ORANGE proteins and their capability to increase provitamin A carotenoids accumulation
Source: PLoS One. 2022 Jan 7;17(1):e0262412. doi: 10.1371/journal.pone.0262412 (PMC8741059; doi:10.1371/journal.pone.0262412)
Supplement: S3 File — Red arrows indicate the presence of a SNP. (PDF) [file pone.0262412.s009.pdf]

```
MePSY1_Ref      ATGACTATAGCGTTACTATGGGTTGCCATTCCCAGTACAGAGGTTTCCAACTCCTTTGGA    60
MePSY1_Seq      ATGACTATAGCGTTACTATGGGTTGCCATTCCCAGTACAGAGGTTTCCAACTCCTTTGGA    60
                ************************************************************

MePSY1_Ref      TTCTTCCATTCGGTTGGGGCTCTAGATTCAGCCAAGTTTGGTTCTGTAGATCGAAGTTTG    120
MePSY1_Seq      TTCTTCCATTCGGTTGGGGCTCTAGATTCAGCCAAGTTTGGTTCTGTAGATCGAAGTTTG    120
                ************************************************************

MePSY1_Ref      ATGTTTAAGAAAAGAGCAAAAAAGGGTATGAACCAGAAATGGAAGTCCAGCACAGTGAAT    180
MePSY1_Seq      ATGTTTAAGAAAAGAGCAAAAAAGGGTATGAACCAGAAATGGAAGTCCAGCACAGTGAAT    180
                ************************************************************

MePSY1_Ref      GTAGATTTGACGAATCCTTGCATAGGATTAGGTAGTGGAAGCAATTTACCTGTAATATCG    240
MePSY1_Seq      GTAGATTTGACGAATCCTTGCATAGGATTAGGTAGTGGAAGCAATTTACCTGTAATATCG    240
                ************************************************************

MePSY1_Ref      AGCATGGTAGCCAGCTCGGCAGGAGAAATGGCCGTCTCGTCGGAAGAGAAGGTATACAAT    300
MePSY1_Seq      AGCATGGTAGCCAGCTCGGCAGGAGAAATGGCCGTCTCGTCGGAAGAGAAGGTATACAAT    300
                ************************************************************

MePSY1_Ref      GTTGTGCTGAAGCAAGCAGCCTTGGTTAAAAAGCAATTGAGGTCTAGTGAGAATCTGGAT    360
MePSY1_Seq      GTTGTGCTGAAGCAAGCAGCCTTGGTTAAAAAGCAATTGAGGTCTAGTGAGAATCTGGAT    360
                ************************************************************

MePSY1_Ref      GCGAAAACAGATATTGCGGTTCCAGGGACTTCGAGCTTGTTGAGCGAAGCTTATGATCGT    420
MePSY1_Seq      GCGAAAACAGATATTGCGGTTCCAGGGACTTCGAGCTTGTTGAGCGAAGCTTATGATCGT    420
                ************************************************************

MePSY1_Ref      TGTGGAGAAGTTTGTGCTGAGTATGCCAAGACATTTTACTTGGGAACTCTGTTAATGACC    480
MePSY1_Seq      TGTGGAGAAGTTTGTGCTGAGTATGCCAAGACATTTTACTTGGGAACTCTGTTAATGACC    480
                ************************************************************

MePSY1_Ref      CCTGAAAGGCGAAGAGCTATCTGGGCAATTTATGTGTGGTGTAGAAGGACAGATGAGCTT    540
MePSY1_Seq      CCTGAAAGGCGAAGAGCTATCTGGGCAATTTATGTGTGGTGTAGAAGGACAGATGAGCTT    540
                ************************************************************

MePSY1_Ref      GTTGACGGACCTAATGCTTCACACATAACACCCACAGCTTTAGATAGATGGGAGGCAAGG    600
MePSY1_Seq      GTTGACGGACCTAATGCTTCACACATAACACCCACAGCTTTAGATAGATGGGAGGCAAGG    600
                ************************************************************

MePSY1_Ref      TTGGAAGATGTTTTCCAAGGTCGTCCTTTTGATATGCTTGATGCTGCTTTATCAGATACT    660
MePSY1_Seq      TTGGAAGATGTTTTCCAAGGTCGTCCTTTTGATATGCTTGATGCTGCTTTATCAGATACT    660
                ************************************************************

MePSY1_Ref      GTTACTAAATTTCCTGTTGATATTCAGCCATTCAAAGATATGATTGAAGGAATGAGGATG    720
MePSY1_Seq      GTTACTAAATTTCCTGTTGATATTCAGCCATTCAAAGATATGATTGAAGGAATGAGGATG    720
                ************************************************************

MePSY1_Ref      GACCTGAAGAAGTCAAGATATAATAACTTTGACGAGCTGTATCTTTACTGTTATTATGTT    780
MePSY1_Seq      GACCTGAAGAAGTCAAGATATAATAACTTTGACGAGCTGTATCTTTACTGTTATTATGTT    780
                ************************************************************

MePSY1_Ref      GCTGGGACAGTTGGATTAATGAGTGTTCCAGTGATGGGCATTGCACCTGAATCACAGGCA    840
MePSY1_Seq      GCTGGGACAGTTGGATTAATGAGTGTTCCAGTGATGGGCATTGCACCTGAATCACAGGCA    840
                ************************************************************

MePSY1_Ref      TCAACTGAGAGCGTTTACAATGCTGCTTTAGCATTAGGAATAGCCAATCAGCTCACCAAC    900
MePSY1_Seq      TCAACTGAGAGCGTTTACAATGCTGCTTTAGCATTAGGAATAGCCAATCAGCTCACCAAC    900
                ************************************************************

MePSY1_Ref      ATACTCAGAGATGTAGGAGAAGATGCACGAAGAGGAAGGATTTATTTACCACAGGACGAG    960
MePSY1_Seq      ATACTCAGAGATGTAGGAGAAGATGCACGAAGAGGAAGGATTTATTTACCACAGGACGAG    960
                ************************************************************

MePSY1_Ref      CTGGCGCAGGCAGGACTTTCAGATGAAGACATATTTGCTGGAGAAGTAACAAATAAGTGG    1020
MePSY1_Seq      CTGGCGCAGGCAGGACTTTCAGATGAAGACATATTTGCTGGAGAAGTAACAAATAAGTGG    1020
                ************************************************************

MePSY1_Ref      AGAAATTTCATGAAGAATCAAATTAAGAGAGCAAGGATGTTCTTCAATGAAGCAGAGAAA    1080
MePSY1_Seq      AGAAATTTCATGAAGAATCAAATTAAGAGAGCAAGGATGTTCTTCAATGAAGCAGAGAAA    1080
                ************************************************************

MePSY1_Ref      GGAGTTACAGAGCTAAGTGCTGCAAGTAGATGGCCGGTGTGGGCATCCTTGCTATTGTAC    1140
MePSY1_Seq      GGAGTTACAGAGCTAAGTGCTGCAAGTAGATGGCCGGTGTGGGCATCCTTGCTATTGTAC    1140
                ************************************************************

MePSY1_Ref      AAGCAAATACTAGACGAGATAGAAGCAAATGATTACAACAACTTCACAGAGAGGGCTTAC    1200
MePSY1_Seq      AAGCAAATACTAGCCGAGATAGAAGCAAATGATTACAACAACTTCACAGAGAGGGCTTAT    1200
                ************* ********************************************* 

MePSY1_Ref      GTGAACAAAGCCAAGAAGCTAGCTTTCTTGCCAATTGCTTATGCAAGATCATTTGTTGGG    1260
MePSY1_Seq      GTGAACAAAGCCCAGAAGCTAGCTTTCTTGCCAATTGCTTATGCAAGATCATTTGTTGGG    1260
                ************ ***********************************************

MePSY1_Ref      TCGTCGAGAGTGTCGCCTCCTTTGGCAAACCCATGA    1296
MePSY1_Seq      TCGTCGAGAGTGTCGCCTCCTTTGGCAAGCCCATGA    1296
                **************************** *******
```

S3 File. Alignment of full lenght of *MePSY1* CDS using Clustal Omega. Red arrows indicate the presence of a SNP
