# Supplementary material for: Characterization of cassava ORANGE proteins and their capability to increase provitamin A carotenoids accumulation
Source: PLoS One. 2022 Jan 7;17(1):e0262412. doi: 10.1371/journal.pone.0262412 (PMC8741059; doi:10.1371/journal.pone.0262412)
Supplement: S4 File — Red arrows indicate the presence of a SNP. (PDF) [file pone.0262412.s010.pdf]

```
MePSY2_Ref      ATGACTGTAGCATTACTATGGGTTGCGATTCCCAGTACAGAGGTCTCCAACTCCTTCGGA    60
MePSY2_Seq      ATGACTGTAGCATTACTATGGGTTGCGATTCCCAGTACAGAGGTCTCCAACTCCTTCGGA    60
                ************************************************************

MePSY2_Ref      TTCTTCCATTCGGTTCGAGTTTTAGATTCATCCAAGTTTGGTTGCGTAGATCGGAATTTG    120
MePSY2_Seq      TTCTTCCATTCGGTTCGAGTTTTAGATTCATCCAAGTTTGGTTGCGTAGATCGGAATTTG    120
                ************************************************************

MePSY2_Ref      ATGTTTAAGGGGAAAGCAAAAAAGGGTAGGAACCAGAAATGGAAGTCAGGATCGGTGAGT    180
MePSY2_Seq      ATGTTTAAGGGGAAAGCAAAAAAGGGTAGGAACCAGAAATGGAAGTCAGGATCGGTGAGT    180
                ************************************************************

MePSY2_Ref      ATAGATTTGAGGAGTACTTGCATAGGTAGCGGACGCAAGCTACCAATAATATCGAGCATG    240
MePSY2_Seq      ATAGATTTGAGGAGTACTTGCATAGGTAGCGGACGCAAGCTACCAATAATATCGAGCATG    240
                ************************************************************

MePSY2_Ref      GTGGCGAGTCACGCAGGAGAAATAGCCATCTCCTCTGAGGAAAAAGTATACAATGTGGTG    300
MePSY2_Seq      GTGGCGAGTCACGCAGGAGAAATAGCCATCTCCTCTGAGGAAAAAGTATACAATGTGGTG    300
                ************************************************************

MePSY2_Ref      CTGAAGCAAGCAGCCTTGGTTAAACAGCAATTAAAGTCTAGTGAAGATCTAGACGTGAAA    360
MePSY2_Seq      CTGAAGCAAGCAGCCTTGGTTAAACAGCAATTAAAGTCTAGTGAAGATCTAGACGTGAAA    360
                ************************************************************

MePSY2_Ref      CCAGATATTGTGCTTCCAGGGACTTTGAGCTTGTTGAGCGAAGCTTACGATCGATGTGGG    420
MePSY2_Seq      CCAGATATTGTGCTTCCAGGGACTTTGAGCTTGTTGAGCGAAGCTTACGATCGATGTGGG    420
                ************************************************************

MePSY2_Ref      GAAGTTTGTGCTGAGTATGCCAAGACTTTTTACTTGGGAACTCTGTTAATGACCCCTGAA    480
MePSY2_Seq      GAAGTTTGTGCTGAGTATGCCAAGACTTTTTACTTGGGAACTCTGTTAATGACCCCTGAA    480
                ************************************************************

MePSY2_Ref      AGGCGAAGAGCTATTTGGGCAATATATGTGTGGTGTAGAAGGACAGATGAGCTTGTTGAT    540
MePSY2_Seq      AGGCGAAGAGCTATTTGGGCAATATATGTGTGGTGTAGAAGGACAGATGAGCTTGTTGAT    540
                ************************************************************

MePSY2_Ref      GGACCTAATGCTTCACACATAACGCCAACAGCTTTAGATAGGTGGGAAGCAAGGTTGGAA    600
MePSY2_Seq      GGACCTAACGCTTCACACATAACGCCAACAGATTTAGATAGGTGGGAAGCAAGGTTGGAA    600
                ******** ********************** ****************************

MePSY2_Ref      GATATGTTTCGAGGTCGTCCCTTTGATATGCTTGATGCTGCTTTATCAGATACAGTTACT    660
MePSY2_Seq      GATATGTTTCGAGGTCGTCCCTTTGATATGCTTGATGCTGCTTTATCAGATACAGTTACT    660
                ************************************************************

MePSY2_Ref      AAATTTCCTGTTGATATTCAGCCATTCAAAGATATGATTGAAGGAATGAGGATGGATCTG    720
MePSY2_Seq      AAATTTCCTGTTGATATTCAGCCATTCAAAGATATGATTGAAGGAATGAGGATGGATCTG    720
                ************************************************************

MePSY2_Ref      AAGAAGTCCAGATATAAGAACTTTGACGAGCTTTATCTTTACTGTTATTATGTTGCTGGG    780
MePSY2_Seq      AAGAAGTCCAGATATAAGAACTTTGACGAGCTTTATCTTTACTGTTATTATGTTGCTGGG    780
                ************************************************************

MePSY2_Ref      ACGGTTGGATTAATGAGTGTTCCAGTGATGGGCATTGCACCTGAATCACAGGCATCAACT    840
MePSY2_Seq      ACGGTTGGATTAATGAGTGTTCCAGTGATGGGCATTGCACCTGAATCACAGGCATCAACT    840
                ************************************************************

MePSY2_Ref      GAGAGTGTTTACAATGCTGCTTTAGCACTAGGAATAGCCAATCAGCTGACCAACATACTC    900
MePSY2_Seq      GAGAGTGTTTACAATGCTGCTTTAGCACTCGGAATAGCCAATCAGCTGACCAACATACTC    900
                ***************************** ******************************

MePSY2_Ref      AGGGATGTCGGAGAGGATGCAAGAAGAGGAAGGATTTATTTACCACAGGATGAATTGGCA    960
MePSY2_Seq      AGGGATGTCGGAGAGGATGCAAGAAGAGGAAGGATTTATTTACCACAGGATGAATTGGCA    960
                ************************************************************

MePSY2_Ref      CAGGCAGGGCTTTCAGATGATGACATATTTGCTGGAAAAGTGACAGACAAATGGAGAAAT    1020
MePSY2_Seq      CAGGCAGGGCTTTCAGATGATGACATATTTGCTGGAAAAGTGACAGACAAATGGAGAAAT    1020
                ************************************************************

MePSY2_Ref      TTCATGAAGAACCAGATTAAAAGAGCAAGAATGTTCTTTAACGAGGCAGAGAAAGGAGTG    1080
MePSY2_Seq      TTCATGAAGAACCAGATTAAAAGAGCAAGAATGTTCTTTAACGAGGCAGAGAAAGGAGTG    1080
                ************************************************************

MePSY2_Ref      ACAGAGCTGAGTGCTGCAAGTAGATGGCCGGTATGGGCATCCTTGCTGCTGTACAGAAGA    1140
MePSY2_Seq      ACAGAGCTGAGTGCTGCAAGTAGATGGCCGGTATGGGCATCCTTGCTGCTGTACAGAAGA    1140
                ************************************************************

MePSY2_Ref      ATACTAGACGAGATAGAAGCAAATGATTACAACAACTTCACAAAGAGGGCTTATGTGAGC    1200
MePSY2_Seq      ATACTAGACGAGATAGAAGCAAATGATTACAACAACTTCACAAAGAGGGCTTATGTGAGC    1200
                ************************************************************

MePSY2_Ref      AAAACCAAGAAGATAGCATCTTTGCCAATTGCATATGCAAGATCATTTGTTGGGCCTTCA    1260
MePSY2_Seq      AAAACCAAGAAGATAGCTTCTTTGCCAATTGCATATGCAAGATCATTTGTTGGGCCTTCA    1260
                ***************** ******************************************

MePSY2_Ref      AGAATGTCATCTCCTGTGACAAAAGCTTGA  1290
MePSY2_Seq      AGAATGTCATCTCCTGTGACAAAAGCTTGA  1290
                ******************************
```

S4 File. Alignment of full lenght of *MePSY2* CDS using Clustal Omega. Red arrows indicate the presence of a SNP.
