# Supplementary material for: Characterization of cassava ORANGE proteins and their capability to increase provitamin A carotenoids accumulation
Source: PLoS One. 2022 Jan 7;17(1):e0262412. doi: 10.1371/journal.pone.0262412 (PMC8741059; doi:10.1371/journal.pone.0262412)
Supplement: S5 File — Red arrows indicate the presence of a SNP. (PDF) [file pone.0262412.s011.pdf]

```
MeOR_X1_Ref      ATGGGAAGTTTGGGCCGAGTCCTGGCGGTTTTGTACCCAAATAAGCTACTATGCTTGTCC   60
MeOR_X1_Seq      ATGGGAAGTTTGGGCCGAGTCCTGGCGGTTTTGTACCCAAATAAGCTACTATGCTTGTCC   60
                 ************************************************************

MeOR_X1_Ref      GGTTCTCACGGTTTACATCACCTGAACTGTAGGTTTAAAGGTTTCGACCGGAAATTAAGC   120
MeOR_X1_Seq      GGTTCTCACGGTTTACATCACCTGAACTGTAGGTTTAAAGGTTTCGACCGGAAATTAAGC   120
                 ************************************************************

MeOR_X1_Ref      TCGAAATGGCGATCTATGGCGTCCGAGTTCGAATCTTCTTCTTTCGCGCCTTCTGTTGAC   180
MeOR_X1_Seq      TCGAAATGGCGATCTATGGCGTCCGAGTTCGAATCTTCTTCTTTCGCGCCTTCTGTTGAC   180
                 ************************************************************

MeOR_X1_Ref      TCTGATTCCACTGATAAAAACGCCGCTGGATTTTGTATCATAGAAGGACCTGAAACGGTT   240
MeOR_X1_Seq      TCTGATTCCACTGATAAAAACGCCGCTGGATTTTGTATCATAGAAGGACCTGAAACGGTT   240
                 ************************************************************

MeOR_X1_Ref      CAAGACTTTGCTAAAATGGAACTGCAGGAAATTCGAGATAACATTCGAAGCCGGCGGAAC   300
MeOR_X1_Seq      CAAGACTTTGCTAAAATGGAACTGCAGGAAATTCGAGATAACATTCGAAGCCGGCGGAAC   300
                 ************************************************************

MeOR_X1_Ref      AAAATTTTCTTGCAGATGGAGGAGGTTCGTAGGCTAAGGATACAACAGAGAATCAAGAGT   360
MeOR_X1_Seq      AAAATTTTCTTGCAGATGGAGGAGGTTCGTAGGCTAAGGATACAACAGAGAATCAAGAGT   360
                 ************************************************************

MeOR_X1_Ref      GCTGAGCTTGGGATTTTAAAGGAAGACCATGAGCATGAGCTTCCTGACTTTCCATCATTC   420
MeOR_X1_Seq      GCTGAGCTTGGGATTTTAAAGGAAGACCATGAGCATGAGCTTCCTGACTTTCCATCATTC   420
                 ************************************************************

MeOR_X1_Ref      ATCCCCTTTTTGCCTCCTTTGAGTGCAGAAAATCTTAAGCTGTACTATGCTACTTGTTTT   480
MeOR_X1_Seq      ATCCCCTTTTTGCCTCCTTTGAGTGCAGAAAATCTTAAGCTGTACTATGCTACTTGTTTT   480
                 ************************************************************

MeOR_X1_Ref      TCTCTTATTGCTGGGATCATCATTTTTGGCGGCCTTTTAGCACCTATTTTGGAAATTAAG   540
MeOR_X1_Seq      TCTCTTATTGCTGGGATCATCATTTTTGGCGGCCTTTTAGCACCTATTTTGGAAATTAAG   540
                 ************************************************************

MeOR_X1_Ref      CTGGGGTTAGGGGGCACGTCATATGCAGATTTTATCCGTTGTGTACATTTGCCTATGCAA   600
MeOR_X1_Seq      CTGGGGTTAGGGGGCACGTCATATGCAGATTTTATCCGTAGTGTACATTTGCCTATGCAA   600
                 *************************************** ********************

MeOR_X1_Ref      TTAAGCCAAGTTGATCCTATAGTGGCTTCATTCTCTGGAGGAGCAGTTGGGGTGATCTCA   660
MeOR_X1_Seq      TTAAGCCAAGTTGATCCTATAGTGGCTTCATTCTCTGGAGGAGCAGTTGGGGTGATCTCA   660
                 ************************************************************

MeOR_X1_Ref      GCCTTGATGGTAGTTGAGATAAACAATGTAAAACAACAAGAACATAAAAGATGCAAATAT   720
MeOR_X1_Seq      GCCTTGATGGTAGTTGAGATAAACAATGTAAAACAACAAGAACATAAAAGATGCAAATAT   720
                 ************************************************************

MeOR_X1_Ref      TGTCTTGGAACTGGGTATCTGGCTTGTGCACGTTGCTCAAGCACTGGATCACTTGTTCTT   780
MeOR_X1_Seq      TGTCTTGGAACTGGGTATCTGGCTTGTGCACGTTGCTCAAGCACTGGATCACTTGTTCTT   780
                 ************************************************************

MeOR_X1_Ref      GTTGAAACAGTTTCAACAGTCAATGGTGGAGATCAACCATTATCCACACCCAAAACAGAA   840
MeOR_X1_Seq      GTTGAAACAGTTTCAACAGTCAATGGTGGAGATCAACCATTATCCACACCCAAAACAGAA   840
                 ************************************************************

MeOR_X1_Ref      AGATGTTCGAATTGTTCAGGATCTGGAAAGGTCATGTGCCCCACATGCCTTTGCACTGGA   900
MeOR_X1_Seq      AGATGTTCGAATTGTTCAGGATCTGGAAAGGTCATGTGCCCCACATGCCTTTGCACTGGA   900
                 ************************************************************

MeOR_X1_Ref      ATGGCTATGGCTAGTGAACACGACCCAAGGATTGACCCCTTTGATTAG      948
MeOR_X1_Seq      ATGGCTATGGCTAGTGAACACGACCCAAGGATTGACCCCTTTGATTAG      948
                 ************************************************
```

S5 File. Alignment of full lenght of *MeOR_X1* CDS using Clustal Omega. Red arrows indicate the presence of a SNP.
