# Supplementary material for: Characterization of cassava ORANGE proteins and their capability to increase provitamin A carotenoids accumulation
Source: PLoS One. 2022 Jan 7;17(1):e0262412. doi: 10.1371/journal.pone.0262412 (PMC8741059; doi:10.1371/journal.pone.0262412)
Supplement: S6 File — Red arrows indicate the presence of a SNP. (PDF) [file pone.0262412.s012.pdf]

```
MeOR_X2_Ref      ATGCTTCACCTCCAACTCCTATGCTTCTCTTGTCTCTATTATCCTCCTTCACCTGATTCC   60
MeOR_X2_Seq      ATACTTCACCTCCAACTCCTATGCTTCTCTTGTCTCTATTATCCTCCATCACCTGATTCC   60
                 ** ******************************************** ************

MeOR_X2_Ref      GATAAACAGTTGCAGATCTTTCTTCGTTTAAGACGAAACAAAAAGTTCTTTGTATCTATA   120
MeOR_X2_Seq      GATAAACAGTTGCAGATCTTTCTTCGTTTAAGACGAAACAAAAAGTTCTTTGTATCTATA   120
                 ************************************************************

MeOR_X2_Ref      TTGTTGTTGCAACAATACGAGACGGAGACTCACAGATTGGGGGTCTCGATTATGGGTAGT   180
MeOR_X2_Seq      TTGTTGTTGCAACAATACGAGACGGAGACTCTCAGATTGGGGGTCTCGATTATGGGTAGT   180
                 ******************************* ****************************

MeOR_X2_Ref      TTGAGCCGTGTTTTGGCGGTTTCTTACCCACATAAACCGCTGTGCCTGTCTGGTCCTCAC   240
MeOR_X2_Seq      TTGAGCCGTGTTTTGGCGGTTTCTTACCCACATAAACCGCTGTGCCTGTCTGGTCCTCAC   240
                 ************************************************************

MeOR_X2_Ref      AGCTTACATCACTCGAACTATAGATTTGTAGATGTCAACCGCAAATCGAGCTCGAAATGG   300
MeOR_X2_Seq      AGCTTACATCACTCGAACTATAGATTTGTAGATGTCAACCGCAAATCGAGCTCGAAATGG   300
                 ************************************************************

MeOR_X2_Ref      CGATCTATGGCGTCTGAGCTTGAATCTTCTTCCTTCTCGCCTTCTATTGACTCTGATTCA   360
MeOR_X2_Seq      CGATCTATGGCGTCTGAGCTTGAATCTTCTTCCTTCTCGCCTTCTATTGACTCTGATTCA   360
                 ************************************************************

MeOR_X2_Ref      ACTGATAAAACCGCCGCCGGGTTTTGTATCATAGAAGGGCCTGAAACGGTTCAAGACTTA   420
MeOR_X2_Seq      ACTGATAAAACCGCCGCCGGGTTTTGTATCATAGAAGGGCCTGAAACGGTTCAAGACTTA   420
                 ************************************************************

MeOR_X2_Ref      TCCAAAATGGAACTGCAAGAAATTCGAGATAATATTCGAAGTCGGCGGAACAAAATTTTC   480
MeOR_X2_Seq      TCCAAAATGGAACTGCAAGAAATTCGAGATAATATTCGAAGTCGGCGGAACAAAATTTTC   480
                 ************************************************************

MeOR_X2_Ref      TTGCATATGGAGGAGGTTCGTAGGCTGAGGATACAACAGAGAATCAAGAGTGCTGAACTT   540
MeOR_X2_Seq      TTGCATATGGAGGAGGTTCGTAGGCTGAGGATACAACAGAGGATCAAGAGTGCTGAACTT   540
                 ***************************************** ******************

MeOR_X2_Ref      GGGATTTTAAAGGAAACGCAAGAGAATGAGCTTCCCAACTTTCCATCATTCATCCCCTTC   600
MeOR_X2_Seq      GGGATTTTAAAGGAAACGCAAGAGAATGAGCTTCCCAACTTTCCATCATTCATCCCCTTC   600
                 ************************************************************

MeOR_X2_Ref      TTGCCTCCTTTGAGTGCAGAAAATCTGAAGTTATACTATGCTACTTGTTTTTCTCTTATT   660
MeOR_X2_Seq      TTGCCTCCTTTGAGTGCAGAAAATCTGAAGTTATACTATGCTACTTGTTTTTCTCTTATT   660
                 ************************************************************

MeOR_X2_Ref      GCTGGGATTATCATTTTTGGTGGCCTTTTAGCACCCACTTTGGAGCTTAAGCTCGGTTTG   720
MeOR_X2_Seq      GCTGGGATTATCATTTTTGGTGGCCTTTTAGCACCCACTTTGGAGCTTAAGCTCGGTTTG   720
                 ************************************************************

MeOR_X2_Ref      GGGGGGACATCATATGAAGATTTTATCCGAAGTGTCCATTTGCCTATGCAGTTGAGTCAG   780
MeOR_X2_Seq      GGGGGGACATCATATGAAGATTTTATCCGAAGTGTCCATTTGCCTATGCAGTTGAGTCAG   780
                 ************************************************************

MeOR_X2_Ref      GTTGATCCTATAGTAGCTTCATTCTCTGGAGGAGCAGTTGGGGTGATCTCAGCATTGATG   840
MeOR_X2_Seq      GTTGATCCTATAGTAGCTTCATTCTCTGGAGGAGCAGTTGGGGTGATCTCAGCATTGATG   840
                 ************************************************************

MeOR_X2_Ref      GTAGTTGAGATAAACAATGTAAAACAACAGGAACATAAAAGATGCAAATATTGTCTTGGA   900
MeOR_X2_Seq      GTAGTTGAGATAAACAATGTAAAACAACAGGAACATAAAAGATGCAAATATTGTCTTGGA   900
                 ************************************************************

MeOR_X2_Ref      ACTGGGTATCTTGCATGTGCTCGTTGCTCAAGCACTGGCGCAGTTGTTCTTATTGAACCA   960
MeOR_X2_Seq      ACTGGGTATCTTGCATGTGCTCGTTGCTCAAGCACTGGCGCAGTTGTTCTTATTGAACCA   960
                 ************************************************************

MeOR_X2_Ref      GTTTCAACAGTCAGTGGTGGGGCTCAGCCACTGTCAGCACCCAAAACAGAAAGATGTTCG   1020
MeOR_X2_Seq      GTTTCAACAGTCAGTGGTGGGGCTCAGCCACTGTCAGCACCCAAAACAGAAAGATGTTCG   1020
                 ************************************************************

MeOR_X2_Ref      AATTGTTCGGGATCTGGAAAGGTGATGTGCCCCACATGCCTTTGCACTGGAATGGCTATG   1080
MeOR_X2_Seq      AATTGTTCGGGATCTGGAAAGGTGATGTGCCCCACATGCCTTTGCACTGGAATGGCTATG   1080
                 ************************************************************

MeOR_X2_Ref      GCAAGTGAACACGACCCGAGGATTGACCCCTTCGATTAG          1119
MeOR_X2_Seq      GCAAGTGAACACGACCCGAGGATTGACCCCTTCGATTAG          1119
                 ***************************************
```

S6 File. Alignment of full lenght of *MeOR_X2* CDS using Clustal Omega. Red arrows indicate the presence of a SNP.
